# Childhood and Adolescent Environmental Risk Factors for Multiple Sclerosis: A Systematic Review With Meta‐Analysis

**DOI:** 10.1111/ene.70398

**Published:** 2025-10-28

**Authors:** Bruno Kusznir Vitturi, Maria Cellerino, Daniele Boccia, Emmanuelle Leray, Jorge Correale, Ruth Dobson, Ingrid van der Mei, Kazuo Fujihara, Matilde Inglese

**Affiliations:** ^1^ Department of Neurology University Hospital Schleswig‐Holstein, Kiel University Campus Kiel Germany; ^2^ Department of Neuroscience, Rehabilitation, Ophthalmology, Genetics, and Mother‐Child Health (DINOGMI) University of Genoa Genoa Italy; ^3^ EHESP, CNRS, Inserm, ARENES UMR 6051, RSMS U 1309 Rennes University Rennes France; ^4^ Department of Neurology Fleni Buenos Aires Argentina; ^5^ Institute of Biological Chemistry and Biophysics CONICET/University of Buenos Aires Buenos Aires Argentina; ^6^ Centre for Preventive Neurology, Wolfson Institute of Population Health Queen Mary University of London London UK; ^7^ Menzies Institute of Medical Research University of Tasmania Hobart Australia; ^8^ Department of Multiple Sclerosis Therapeutics, Fukushima Medical University School of Medicine and Multiple Sclerosis and Neuromyelitis Optica Center Southern TOHOKU Research Institute for Neuroscience Koriyama Japan

**Keywords:** demyelinating diseases, environmental exposure, multiple sclerosis, risk factors

## Abstract

**Background:**

We aimed to provide updated evidence from the current literature regarding pediatric environmental factors associated with the risk of developing multiple sclerosis (MS).

**Methods:**

Articles were searched in PubMed, SciVerse ScienceDirect, and Web of Science. We included all clinical studies assessing the occurrence of MS at any age in association with the exposure to any environmental risk factor during childhood or adolescence. The main outcome was the occurrence of MS. The quality assessment was performed with the critical appraisal checklist for case–control studies. Pooled unadjusted effect sizes (OR) were calculated and reported with a 95% CI from random‐effects meta‐analysis.

**Results:**

The review included 87 studies conducted across 20 countries. The studies analyzed diverse environmental risk factors, including infections, vaccinations, tobacco exposure, body mass index, and other pediatric exposures. EBV infection showed a significant positive association with MS risk (ES = 2.38, 95% CI = 1.80–3.15). Breastfeeding showed limited protective associations, and various adverse social experiences like bullying and sexual abuse were linked to increased MS risk. Active smoking during childhood/adolescence and obesity during these periods were associated with higher MS risk, while normal body mass index was protective. Antibiotic and chemical exposures, as well as vitamin D deficiency, were linked to higher MS risk. The review highlighted substantial heterogeneity and identified publication bias in studies on infections and vaccinations.

**Conclusions:**

Environmental risk factors for MS are important during childhood and adolescence. The first 20 years are a key window for prevention and should be seen as an opportunity.

## Graphical abstract

The review analyzed 87 studies from 20 countries on environmental risk factors for MS. EBV infection increased MS risk, while breastfeeding showed limited protection. Adverse social experiences, active smoking, and obesity in childhood/adolescence raised risk, whereas normal BMI was protective. Antibiotic and chemical exposures, along with vitamin D deficiency, were also linked to higher MS risk.

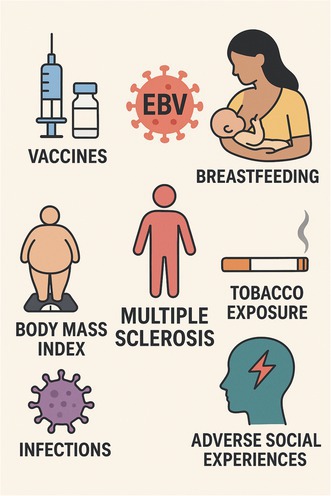

## 1 Introduction

Multiple sclerosis (MS) is a chronic inflammatory, demyelinating, and neurodegenerative disease of the central nervous system (CNS) [1]. While the typical age of MS onset is between 20 and 40 years, about 2% to 10% of patients experience their initial demyelinating event during childhood or adolescence [2, 3, 4]. The exact etiology of MS remains unknown, but the most widely accepted hypothesis suggests that environmental triggers, acting on a genetically predisposed substrate, activate an autoimmune response that leads to CNS damage [1]. Early‐life exposures likely contribute to MS risk, and there may be less bias epidemiologically in studying children.

Literature suggests that lifestyle and environmental factors during infancy and adolescence are crucial in MS physiopathology, with childhood being a sensitive period for disease development [3, 5, 6, 7]. Some factors can be relevant enough to trigger the disease in the first two decades of life. For instance, factors such as lack of breastfeeding, infections (including Epstein–Barr virus), vitamin D deficiency, obesity, maltreatment or sexual abuse, and tobacco exposure (active or passive smoking) during childhood have all been proposed as contributors to the development of pediatric‐onset MS [8, 9, 10]. Additionally, it is well known that the duration of exposure to an environmental risk factor plays a determining role in the causal relationship between these findings and the incidence of chronic neurological diseases [11]. While numerous studies have examined various risk factors, no meta‐analysis has been conducted to synthesize the existing evidence and provide the highest level of evidence. In this view, we decided to conduct the first review with meta‐analysis to provide updated evidence from the current literature on pediatric environmental factors involved in MS‐associated risk.

## 2 Methods

### 2.1 Study Protocol

The present systematic review was designed and reported following the Preferred Reporting Items for Systematic reviews and Meta‐Analysis (PRISMA) guideline [12]. The protocol was registered in the international prospective register of systematic reviews (PROSPERO) with the registration number CRD42023471073.

### 2.2 Data Sources, Search Strategy, and Study Selection

Studies were searched in PubMed, SciVerse ScienceDirect, and Web of Science, with the detailed search strategy presented in the supporting material (Appendix S1). References were managed in Zotero (Corporation for Digital Scholarship—version 6.0.27), which identifies duplicates and allows the researcher to remove them after a second review. After deduplication, titles and abstracts were screened to assess potential relevance, excluding those that did not fit the topic. For all retrieved studies, two independent, previously trained researchers (BKV and MC) reviewed the full texts to determine eligibility according to preestablished inclusion and exclusion criteria, remaining blinded to each other's decisions. In the event of conflicting opinions, a senior researcher (MI) was consulted to promote discussion and reach a consensus.

The inclusion criteria were defined using the acronym PECOS (patients, exposure, comparator, outcomes, study design). We included any original peer‐reviewed article that included adult or pediatric patients diagnosed with MS and whose characteristics were compared with individuals who did not develop the disease. The diagnosis of MS must be based on current standard guidelines or confirmed by a neurologist. All research subjects had to have been exposed to environmental risk factors during childhood and/or adolescence that were potentially related to the development of MS—this criterion must be clear in the article. We defined childhood as the period that extends from birth up to 10years of age, and adolescence as the period that follows childhood and lasts until 19 years of age [13]. The outcome of the studies must have been the occurrence of MS at any age, and the absolute numbers of cases and controls in each exposure category must have been described or provided. Outcomes that included isolated demyelinating events not leading to a specific diagnosis or other demyelinating diseases, such as neuromyelitis optica, acute disseminated encephalomyelitis, and myelin oligodendrocyte glycoprotein antibody‐associated disease (MOGAD) were not accepted. There were no time restrictions. Articles written in English, Spanish, French, Italian, and Portuguese were accepted. Eligible studies could be case–control studies or nested case–control studies (cohort studies). Articles conceived as reviews, clinical trials, conference abstracts, letters to the editor, expert opinions, commentaries, case reports, case series, and editorials were excluded. When more than one article reported from the same cohort/data set and addressed the same variables, the most recent paper was selected, and the other(s) excluded. Moreover, studies that investigated the onset of MS in subjects with an underlying demyelinating event (e.g., optic neuritis) were excluded.

Exposure to any biological agent was defined as serological evidence of contact with the pathogen if measured during childhood or adolescence, or as an official clinical record indicating that the infection occurred during this period. Vaccination status was accepted if self‐reported or documented in any vaccination record or certificate. Tobacco exposure was classified as either active smoking or passive smoking, defined as the inhalation of tobacco smoke by individuals other than the smoker. Breastfeeding at any stage of lactation was considered a potential risk factor for the child. Exposure to other environmental risk factors cited in the literature was considered valid if self‐reported by the research participant for any time prior to the MS diagnosis. The specifics of exposure measurement in each study were critically analyzed during the quality assessment process.

### 2.3 Data Extraction and Synthesis

Data were tabulated in an Excel spreadsheet. In addition to the number of cases and controls with MS, data on the first author, year of publication, country, sample size, age (mean and standard deviation), proportion of female, mean disease duration, and mean age of disease onset were extracted and recorded. For articles lacking essential data required for the quantitative analysis, we promptly contacted the corresponding authors by email to obtain the necessary information. If contact failed, the study was excluded from the analysis. When a multicenter study presented results for each country, the information was separated and treated individually. No automated data extraction software was used. All extracted data were double‐checked one month after the initial extraction to optimize reliability and minimize the risk of bias. The quality assessment was performed with the critical appraisal checklist, developed and validated by the Joanna Briggs Institute according to each study design. This step was also carried out by two independent and previously trained researchers (BKV and MC), always considering the opinion of a third researcher (MI) in case of discrepancy. Data were synthesized in a supplementary table and analyzed after grouping studies that investigated the same category of environmental risk factors.

## 3 Statistical Analysis

All the information was synthesized qualitatively and quantitatively (meta‐analysis). We used the random‐effects model based on the binomial distribution to calculate the pooled effect sizes (ES) regarding the risk of developing MS according to each potential risk factor. We identified a priori potential variables that could be associated with the estimates—age, sex, educational level, disease duration, progressive MS phenotype, and the EDSS. Potential influences on pooled estimates were investigated using subgroup analyses and meta‐regression. Meta‐regression was performed whenever there were sufficient study data on the moderators for this type of analysis. Between‐study heterogeneity was assessed using the *I* ^*2*^ statistic and visually inspecting the forest plot. *I* ^*2*^ more than 75% was regarded as substantial heterogeneity [14]. We investigated the existence of publication bias using Egger's linear regression test [15] and with the visual inspection of the funnel plots. A two‐sided *p* < 0.05 was considered statistically significant. All statistical analyses were performed using STATA/BE v. 18.0 (StataCorp, College Station, TX, USA).

## 4 Results

**FIGURE 1:**
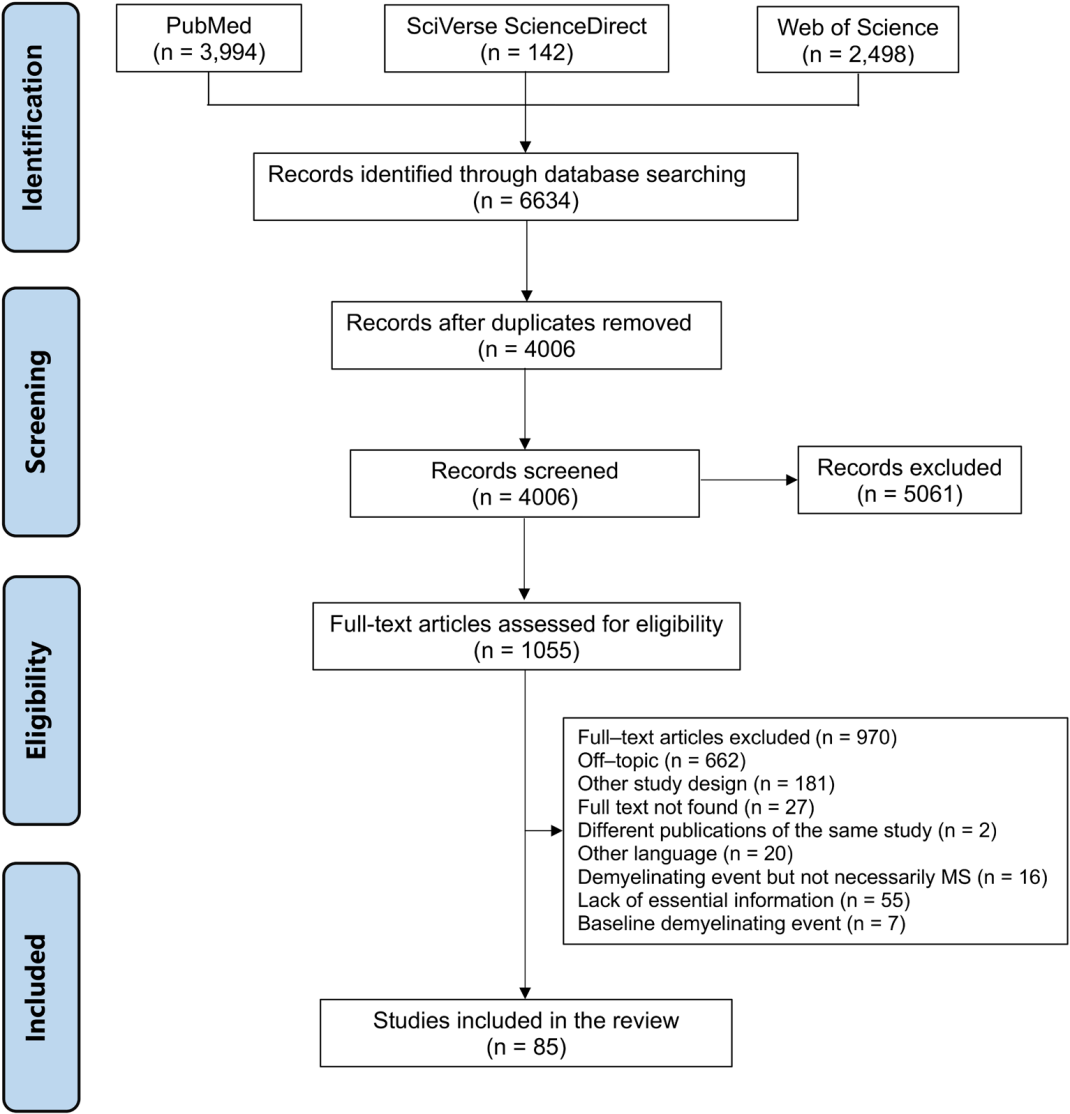
PRISMA flowchart.

A total of 6634 articles matched the search terms. After removing duplicates, 4006 articles were screened by reviewing the titles, abstracts, and full texts. Ultimately, 85 articles met all the eligibility criteria and were included in the review (Figure 1). The studies were conducted in 20 countries across four continents (Appendix S2). The median publication year of the studies was 2016. The average age of PwMS ranged from 9.3 to 55.2 years, with the proportion of women varying from 26.6% to 100%. The mean disease duration ranged from 1.0 to 18.2 years, while the mean age of disease onset varied between 12.3 and 39.5 years. The reporting quality of most articles was deemed satisfactory (Appendix S3), with the most common flaws being the failure to assess the duration of exposure and potential confounding factors. A total of nine groups of environmental risk factors were investigated as potential risk factors for MS. The article was drafted according to the PRISMA checklist (Appendix S4).

**FIGURE 2:**
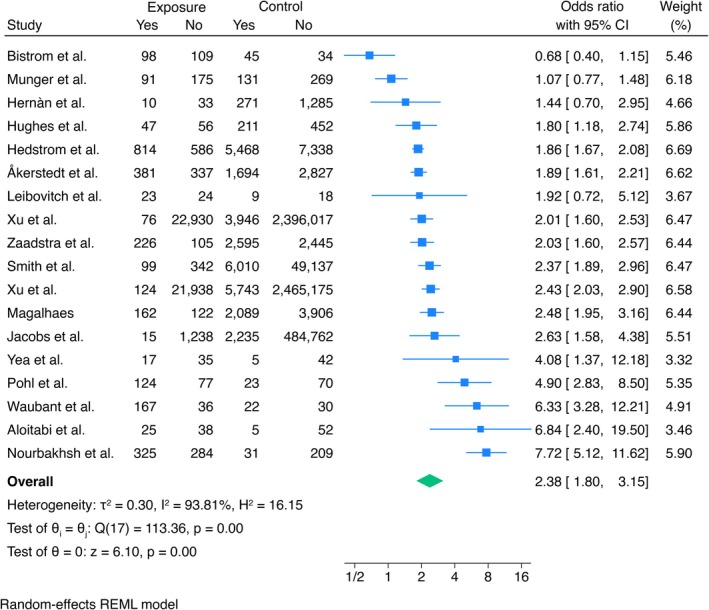
Meta‐analysis of the association between EBV and MS.

Eighteen studies included in the review examined the role of previous EBV infection in the risk of developing MS. The pooled ES indicated a positive association between EBV infection and the occurrence of MS (ES = 2.38, 95% CI = 1.80–3.15) (Figure 2). In the meta‐regression analyses, both disease duration (*p* < 0.001) and disease onset (*p* < 0.001) were negatively associated with the ES. Age and sex did not show statistically significant associations with the outcome (*p* = 0.482 and *p* = 0.235, respectively).

**FIGURE 3:**
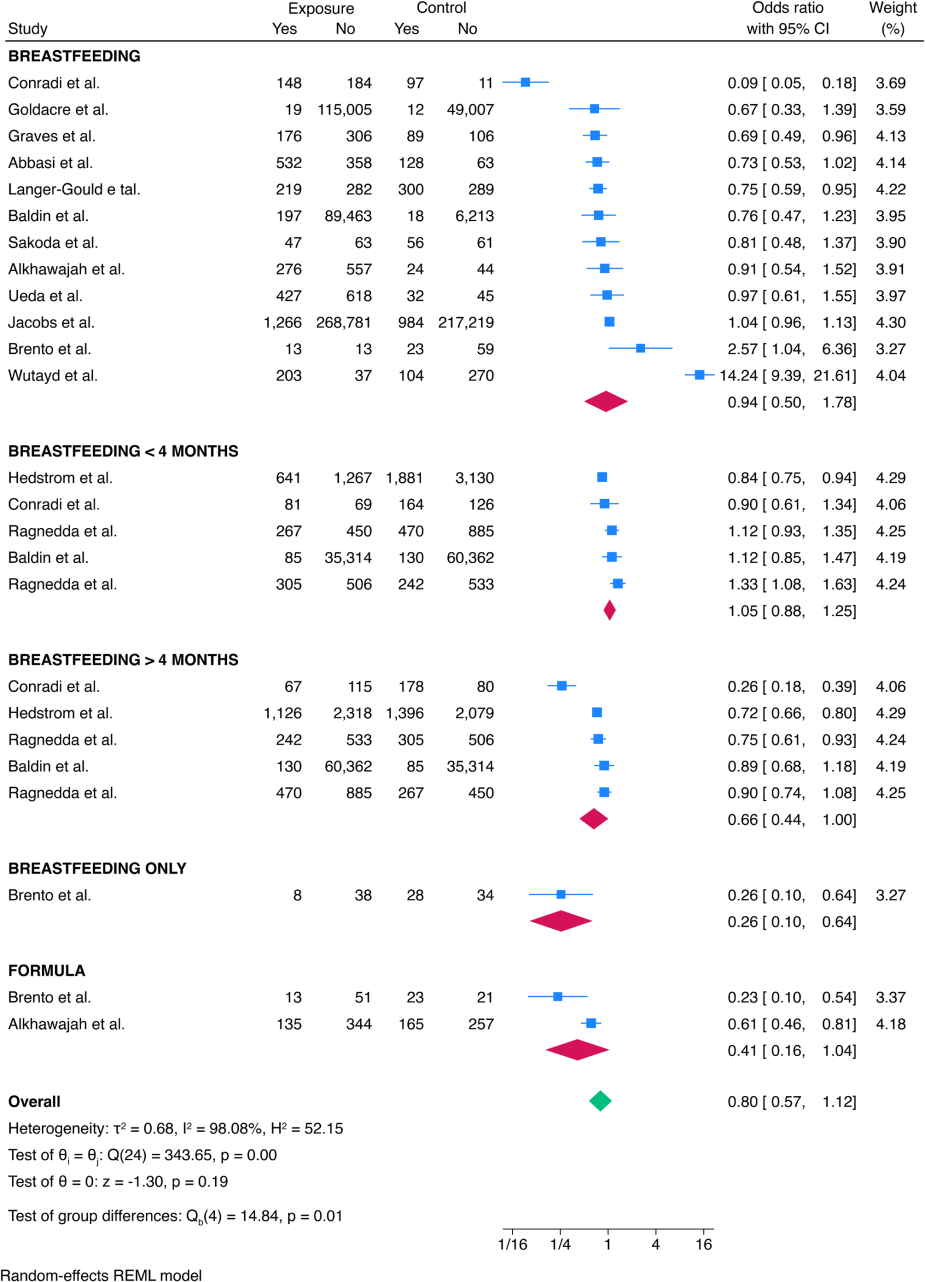
Meta‐analysis of the association between breastfeeding and MS.

The relationship between breastfeeding and MS was extensively explored in the literature (Figure 3). A single study found a positive association between exclusive breastfeeding and a reduced risk of MS (ES = 0.26, 95% CI = 0.10–0.64). However, the pooled ES did not reveal a significant association between MS and breastfeeding in general (ES = 0.94, 95% CI = 0.50–1.78), breastfeeding for less than 4 months (ES = 1.05, 95% CI = 0.88–1.25), breastfeeding for more than 4 months (ES = 0.66, 95% CI = 0.44–1.00), or the use of infant formula (ES = 0.41, 95% CI = 0.16–1.04). Age and sex did not influence the ES (*p* = 0.496 and *p* = 0.642, respectively).

**FIGURE 4:**
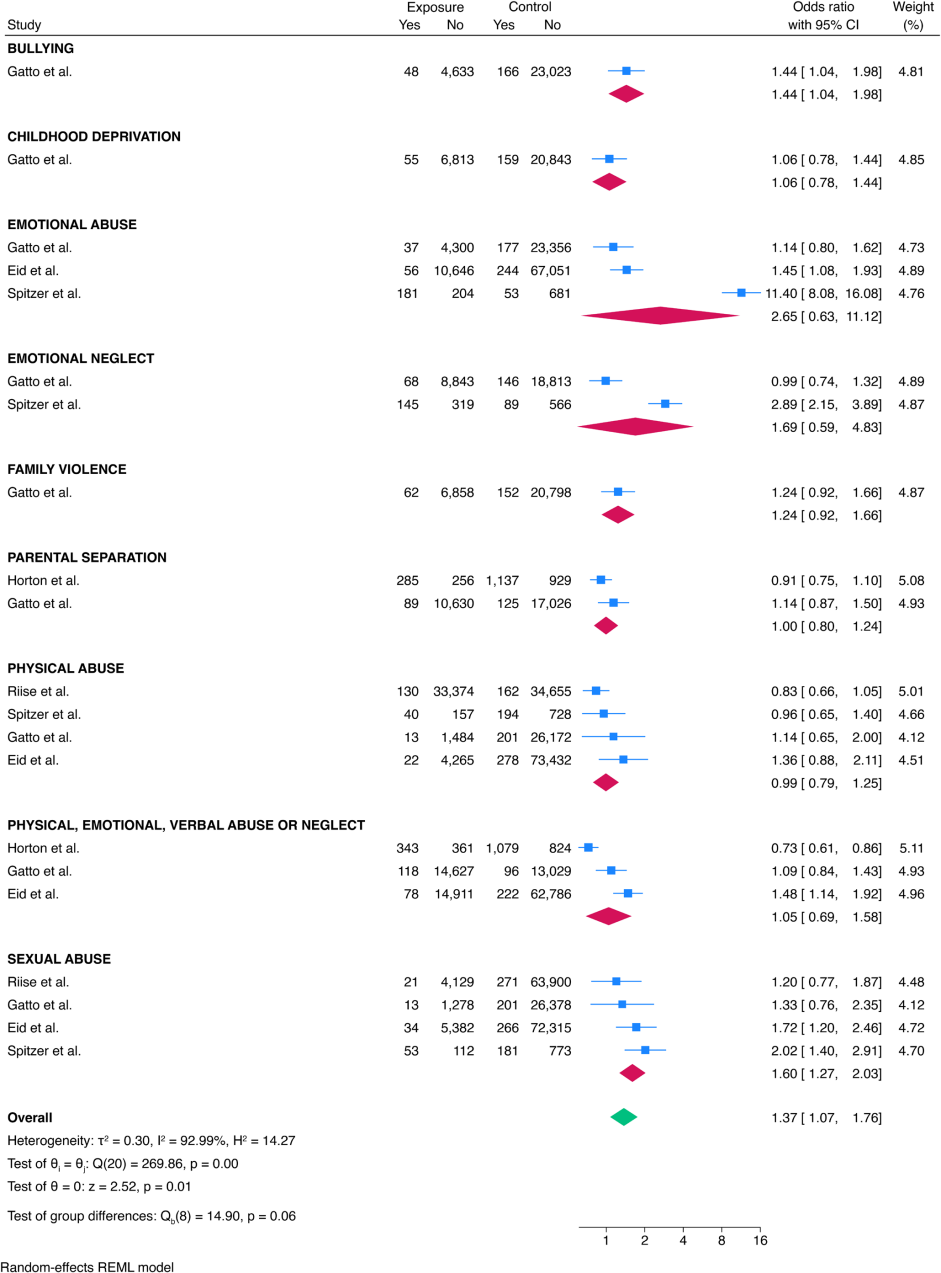
Meta‐analysis of the association between adverse social experiences and MS.

Various types of adverse social experiences were also studied in relation to MS risk (Figure 4). The overall pooled ES was 1.37 (95% CI = 1.07–1.76). Bullying and sexual abuse were significantly associated with an increased risk of developing MS (ES = 1.44, 95% CI = 1.04–1.98, and ES = 1.60, 95% CI = 1.27–2.03, respectively). No significant associations were found between MS and childhood deprivation (ES = 1.06, 95% CI = 0.78–1.44), emotional abuse (ES = 2.65, 95% CI = 0.63–11.12), emotional neglect (ES = 1.69, 95% CI = 0.59–4.83), family violence (ES = 1.24, 95% CI = 0.92–1.66), parental separation (ES = 1.00, 95% CI = 0.80–1.24), physical abuse (ES = 0.99, 95% CI = 0.79–1.25), or physical, emotional, or verbal abuse or neglect (ES = 1.05, 95% CI = 0.69–1.58). Age was found to positively influence the ES in the meta‐regression (*p* = 0.007), while sex and the prevalence of RRMS, PPMS, and SPMS were not significantly associated with the results (*p* = 0.104 for each).

The articles included in the review investigated the potential influence of nine different pediatric infections on the risk of MS, with eight caused by viruses and one by bacteria (Appendix S5). The viral infections studied were varicella‐zoster virus, cytomegalovirus, HHV‐6, HSV‐1/2, measles virus, mumps virus, rubella virus, and variola virus (smallpox), while the bacterial infection was caused by *Bordetella pertussis* . Smallpox was negatively associated with the likelihood of developing MS (ES = 0.62, 95% CI = 0.42–0.91). This ES derived from a single study. No statistically significant association was found for any other pathogens. Age, sex, and disease duration were not significantly associated with the ES (*p* = 0.946, *p* = 0.054, *p* = 0.833, respectively). Later disease onset was positively associated with an increased risk of MS (*p* < 0.001).

Nine vaccines were studied as potential childhood risk factors for MS (Appendix S6). Overall, the analysis of each subgroup included 1 to 4 published studies. Data from a single study that reported cases and controls exposed to diphtheria immunization allowed the calculation of an ES = 4.69 (95% CI = 2.64–8.31). Similarly, the rubella vaccine was positively associated with an increased risk of MS (ES = 2.15, 95% CI = 1.57–2.94). No other vaccines were significantly associated with MS. Meta‐regression was conducted for age and sex, but neither moderator was significantly associated with the pooled ES (*p* = 0.77 for both).

Studies described two types of tobacco exposure during childhood and/or adolescence as potential risk factors for MS: active smoking and passive smoking. Active smoking during these life periods was associated with a higher likelihood of developing MS (ES = 2.60, 95% CI = 1.30–5.18) (Appendix S7). No statistically significant association was observed with passive smoking. Meta‐regression analysis was performed considering age, sex, and disease onset, but none of these moderators showed a significant relationship with the effect size (*p* = 0.94, *p* = 0.49, *p* = 0.06, respectively).

**FIGURE 5:**
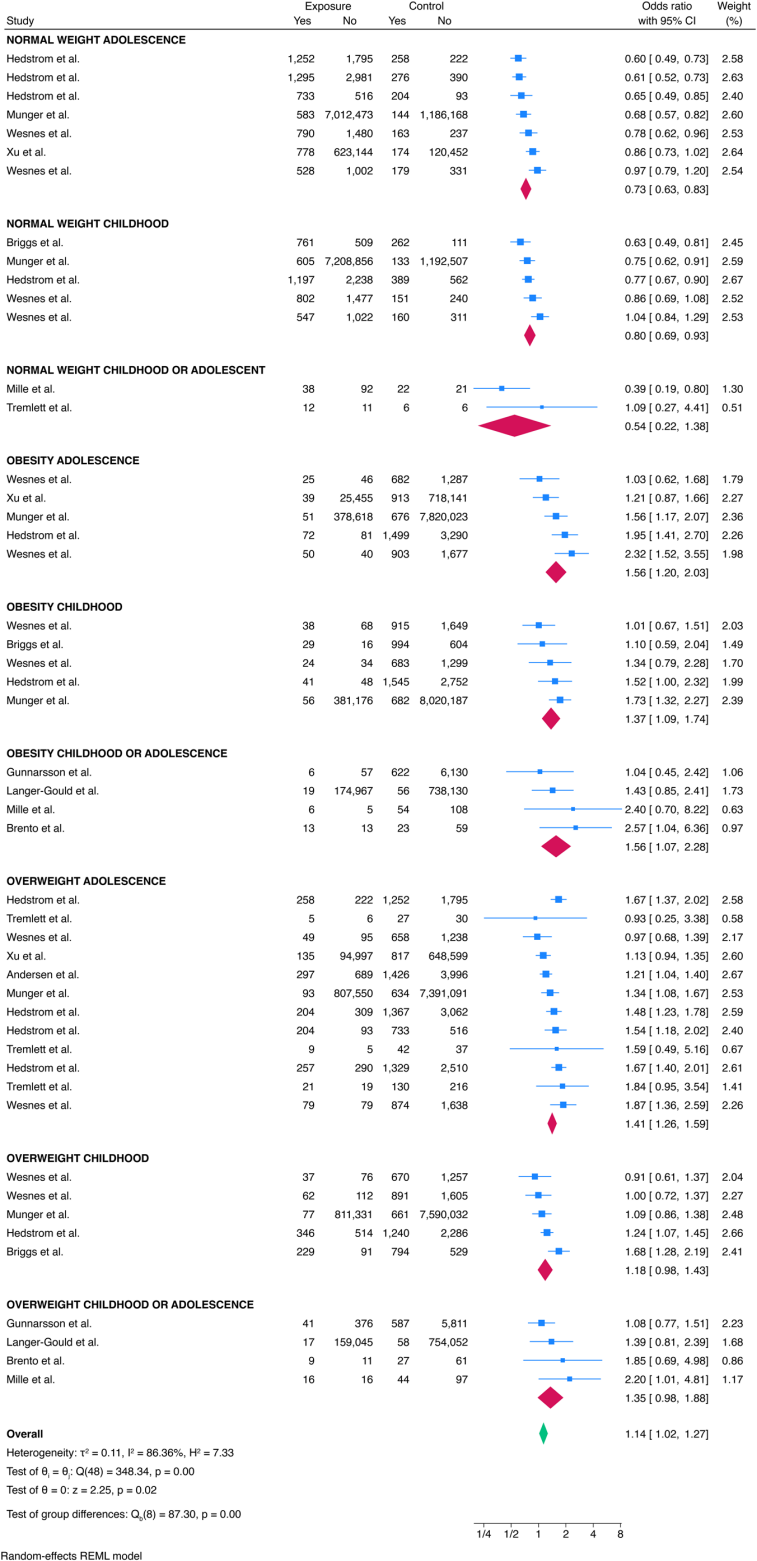
Meta‐analysis of the association between BMI and MS.

Body mass index (BMI) was examined in the literature by categorizing it into normal BMI, overweight, and obesity (Figure 5). The studies focused on these categories during childhood, adolescence, or both periods (not specified). Normal BMI during childhood and adolescence was found to be a protective factor against the risk of developing MS later in life (ES = 0.73, 95% CI = 0.63–0.83). Conversely, overweight during adolescence was associated with increased odds of being diagnosed with MS (ES = 1.41, 95% CI = 1.26–1.59). The risk was also significantly higher for individuals diagnosed as obese during childhood (ES = 1.37, 95% CI = 1.09–1.74), adolescence (ES = 1.56, 95% CI = 1.20–2.03), or during either period (ES = 1.56, 95% CI = 1.07–2.28). No significant associations were found between overweight during childhood and the risk of developing MS. In the meta‐regression analyses, age and sex were not significantly associated with the effect size (*p* = 0.980 and *p* = 0.200, respectively).

Other potential pediatric environmental risk factors, less frequently reported in the literature, included alcohol consumption, antibiotic exposure, chemical exposure, pet exposure, excessive sodium intake, and vitamin D status (Appendix S8). Apart from pet exposure, each of these categories was supported by only one or two studies. An increased risk of MS was observed with antibiotic exposure during the first two years of life (ES = 1.32, 95% CI = 1.25–1.39), exposure to plant insect or disease control products (ES = 1.31, 95% CI = 1.84–2.57), and weed control products (ES = 1.38, 95% CI = 0.56–1.22). Vitamin D deficiency was also associated with a higher ES (ES = 1.30, 95% CI = 1.05–1.62). No significant associations were found between the other variables in this group and the ES. Age and sex had no influence on these analyses (*p* = 0.219 and *p* = 0.487, respectively).

Overall, there was significant heterogeneity across all analyses. As for publication bias, it was detected only in the analyses of the influence of previous infections and vaccination on the risk of developing MS (*p* = 0.010 in both cases). No publication bias was found in the other meta‐analysis (Appendix S9).

## 5 Discussion

To the best of our knowledge, this is the first systematic review and meta‐analysis to examine environmental risk factors during childhood and adolescence associated with MS. Numerous environmental factors have been linked to this association, and it's important to recognize that many of these risk factors are still highly prevalent today. Childhood and adolescence are critical periods for health promotion and the prevention of chronic diseases, including MS.

EBV infection during childhood and/or adolescence is directly associated with MS. There is already substantial evidence of this link from studies involving adults or individuals with positive EBV serologies but no known history of primary infection [16]. This meta‐analysis is the first to show that EBV plays a role in the pathophysiology of MS during the first two decades of life. Current research recognizes EBV as both a trigger and a driver of MS. Persistent EBV infection can lead to molecular mimicry and give B cells a survival advantage, potentially priming latent autoimmunity. Another theory suggests that direct CNS infection could contribute to MS etiology [17, 18].

The findings of this review also highlight the importance of exclusive maternal breastfeeding. Breastfeeding is one of the most impactful early‐life interventions, associated with the prevention of various chronic diseases [19]. Breast milk is rich in nutrients and immune factors that can protect infants against autoimmune conditions [20]. Our study shows that exclusive breastfeeding lowers the risk of developing MS. This protective effect was not observed when breastfeeding was supplemented with formula or when formula alone was used, in line with the WHO's recommendation to prioritize exclusive breastfeeding whenever possible [13].

Several biological agents have already been associated with demyelinating events [21]. However, our review found that most pediatric infections are not linked to MS. Not all biological agents cause neuroinflammation and demyelination, as the interaction between an infectious agent and the CNS is complex, and demyelination triggered by infection also depends on individual susceptibility [21, 22, 23]. Additionally, the immune response and clinical manifestations following viral exposure can vary with the patient's age. For example, while adult studies suggest that HHV‐6 and VZV may increase the risk of MS, our findings did not show an association between these viruses and MS development in children [24, 25]. In some cases, children may be less likely to seroconvert, complicating the establishment of causality through epidemiological studies alone [26]. In the case of smallpox, we did find a link to altered MS risk, but the evidence is based on a single, outdated study with notable quality limitations. Moreover, given that smallpox has been eradicated in most parts of the world, it seems unlikely to have influenced the current rise in MS prevalence [27].

Demyelination can potentially be a side effect of certain vaccines [28]. Given that many vaccines are administered during childhood and adolescence, our review found no substantial evidence of a clear association between immunization and MS. This aligns with the observation that neurological side effects from currently available vaccines are rare [29]. While rubella and diphtheria vaccines showed a positive association with MS risk, the evidence is limited and comes from studies with significant methodological flaws.

Tobacco exposure during childhood and adolescence has been identified as a significant risk factor for MS. Smoking and secondhand smoke exposure in children are associated with various negative outcomes, including sudden infant death syndrome, asthma, infections, and increased cancer risk [30]. Early tobacco exposure can also impair brain development and cause neuroanatomical abnormalities [31]. These results are consistent with evidence showing that smoking is a major risk factor for MS in adulthood [32]. While the exact molecular mechanisms connecting smoking to MS remain unclear, smokers have shown irregularities in T‐cell function, along with compromised humoral and cell‐mediated immune responses [33, 34].

Childhood and adolescent obesity were also found to be risk factors for MS. Obesity is recognized as a pro‐inflammatory state that induces several alterations in the CNS [35], including white matter lesions caused by inflammation [36]. The link between adult obesity and MS is well‐documented [37], and childhood represents a critical window for preventing obesity and its associated comorbidities later in life. Obese children and adolescents are at higher risk of becoming obese adults [38], potentially prolonging and exacerbating the harmful effects of excess weight in susceptible individuals. In contrast, maintaining a healthy weight appears to protect against MS, reinforcing the importance of a healthy lifestyle in the epidemiology of MS, as suggested by previous studies [39].

Children are often exposed to a range of adverse social experiences, with the prevalence of adverse childhood experiences (ACEs) reaching as high as 40% [40]. The negative health impacts of these experiences are well established. We demonstrated that age is associated with the risk of MS in children who are victims of ACEs. Indeed, young children may lack the cognitive and psychological capacity to cope with ACEs. ACEs have been linked to chronic diseases such as depression, cardiovascular disease, diabetes, and cancer [41]. Additionally, early‐childhood deprivation has been associated with alterations in adult brain structure, even when environmental conditions improve later on [42]. Our study provides strong evidence that ACEs can also increase the risk of MS. In particular, bullying and sexual abuse seem to significantly elevate MS risk, while other types of adverse experiences did not show a statistically significant association, possibly due to a lack of studies or objective measures linking them to MS.

Preliminary evidence suggests that early exposure to antibiotics, disease control products, and weed control products may be associated with MS risk. Likewise, pesticides and chemicals used in agriculture have been proposed as potential MS risk factors in occupational settings [43]. However, the evidence linking these chemical exposures to MS during childhood and adolescence remains preliminary.

While research on vitamin D deficiency as a childhood MS risk factor is limited, our review confirms that it is indeed a risk factor during this period. There is extensive evidence that vitamin D deficiency plays a key role in MS pathophysiology in adults [44]. It would be interesting to investigate if supplementation of vitamin D during childhood could mitigate MS risk, for example. Moreover, it is important to note that vitamin D levels may naturally vary across racial and ethnic groups in a pattern that does not necessarily correspond to MS susceptibility. Therefore, our findings should be validated through further studies in the context of precision medicine.

This review does have limitations that must be acknowledged, many of which are inherent to the design of any meta‐analysis. First, meta‐analyses may not account for all confounding variables across studies, potentially leading to results that do not fully reflect causal relationships. Specifically, this approach does not allow us to draw conclusions about the interrelationships between environmental risk factors and MS risk. Second, the quality of the included studies can affect the accuracy of the results. Third, meta‐analyses combine data from diverse populations, settings, and methodologies, which may limit the generalizability of the findings to specific groups or contexts. Additionally, we emphasize that many studies did not assess the duration or degree of exposure, which should be a focus in future longitudinal cohort studies. Finally, many studies were published before MOGAD was widely recognized, meaning some patients classified as having MS could have tested positive for MOG‐IgG.

## 6 Conclusion

Environmental risk factors of MS do play an important role during childhood and adolescence. The first two decades of life should be regarded as a golden period for the prevention of MS and viewed as an invaluable opportunity. Furthermore, this phase offers a critical chance to optimize health behaviors that may influence the risk of developing MS. Therefore, further studies should be focused on environmental risk factors of MS during this period.

## Author Contributions

Bruno Kusznir Vitturi had full access to all the data in the study and takes responsibility for the integrity of the data and the accuracy of the data analysis. Concept and design: Bruno Kusznir Vitturi and Matilde Inglese. Acquisition and analysis of data: Bruno Kusznir Vitturi, Maria Cellerino, and Daniele Boccia. Interpretation of data: Bruno Kusznir Vitturi, Daniele Boccia, Maria Cellerino, Ruth Dobson, Ingrid van der Mei, Emmanuelle Leray, Kazuo Fujihara, Jorge Correale, and Matilde Inglese. Drafting of the manuscript: Bruno Kusznir Vitturi, Daniele Boccia, Maria Cellerino, Ruth Dobson, Ingrid van der Mei, and Emmanuelle Leray. Critical revision of the manuscript for important intellectual content: Bruno Kusznir Vitturi, Daniele Boccia, Maria Cellerino, Ruth Dobson, Ingrid van der Mei, Emmanuelle Leray, Kazuo Fujihara, Jorge Correale, and Matilde Inglese. Statistical analysis: Bruno Kusznir Vitturi. Supervision: Matilde Inglese.

## Conflicts of Interest

The authors declare no conflicts of interest.

## Supporting information


**Appendices S1–S9.** Supporting Information.

## Data Availability

The data that support the findings of this study are available from the corresponding author upon reasonable request.
