# Supplementary material for: Childhood and Adolescent Environmental Risk Factors for Multiple Sclerosis: A Systematic Review With Meta‐Analysis
Source: Eur J Neurol. 2025 Oct 28;32(11):e70398. doi: 10.1111/ene.70398 (PMC12560250; doi:10.1111/ene.70398)
Supplement: Supplementary file 1 — Appendices S1–S9. Supporting Information. [file ENE-32-e70398-s001.docx]

**eAppendix 1. Detailed search strategy.**

| **PubMed** | ("children" OR “neonatal” OR “newborn” OR “maternal” OR “infant” OR “perinatal” OR “pediatrics” OR “paediatrics” OR “child” OR “childhood” OR “infancy” OR “adolescence” OR “adolescent”) AND (“multiple sclerosis” OR “clinically isolated syndrome” OR “demyelinating”) AND (“risk factor” OR expos* OR “environmental” OR “cause” OR epidemiolog* OR prevent* OR “etiology”) |
| --- | --- |
| **SciVerse Science Direct** | Title, abstract, keywords: ("children" OR “childhood” OR “neonatal” OR “infancy” OR “adolescence”) AND (“multiple sclerosis”) AND (“risk factor” OR “exposure”) |
| **Web of Science** | ("children" OR “neonatal” OR “newborn” OR “maternal” OR “infant” OR “perinatal” OR “pediatrics” OR “paediatrics” OR “child” OR “childhood” OR “infancy” OR “adolescence” OR “adolescent”) AND (“multiple sclerosis” OR “clinically isolated syndrome” OR “demyelinating”) AND (“risk factor” OR expos* OR “environmental” OR “cause” OR epidemiolog* OR prevent* OR “etiology”) |

**eAppendix 2. Detailed characteristics of included studies**

| **Authors** | **Year** | **Country** | **N_cases** | **N_controls** | **Age, mean (SD)** | **Sex (%)** | **Disease duration, mean (SD)** | **Disease onset, mean (SD)** | **Exposure** |
| --- | --- | --- | --- | --- | --- | --- | --- | --- | --- |
| Abbasi et al. [1] | 2017 | Iran | 660 | 421 |  |  |  |  | Breastfeeding |
| Abdollahpour et al. [2] | 2021 | Iran | 547 | 1057 | 31.3 (9.3 | 401 |  |  | Tobacco exposure |
| Ahlgren et at. [3] | 2009 | Sweden | 627 | 3917 |  |  |  | 26.4 (6.7) | Vaccines |
| Åkerstedt et al. [4] | 2023 | Sweden | 2075 | 3164 |  | 1502 |  |  | Epstein-baar virus |
| Alkhawajah et al. [5] | 2021 | Saudi Arabia | 300 | 601 | 34.8 (9.2) | 89 |  |  | Breastfeeding |
| Aloitabi et al. [6] | 2004 | Kuwait | 30 | 90 |  |  | 1.4 (1.7) | 12.7 (4.6) | Previous infections |
| Ammitzboll et al. [7] | 1973 | Denmark | 89 | 69 |  |  |  |  | Others |
| Andersen et al. [8] | 2019 | Denmark | 1723 | 4685 | 42.8 (9.5) | 1210 (18.8) |  |  | Tobacco exposure |
| Bager et al. [9] | 2004 | Denmark | 455 | 1801 |  |  |  |  | Previous infections |
| Baldin et al. [10] | 2021 | Norway | 215 | 95676 |  |  |  |  | Breastfeeding |
| Bansil et al. [11] | 1997 | India | 56 | 91 | 34.1 (8.0) | 35 | 6.5 (NA) |  | Previous infections |
| Bistrom et al. [12] | 2021 | Sweden | 143 | 143 |  | 563 |  |  | Previous infections |
| Bourne et al. [13] | 2017 | USA | 271 | 418 |  | 165 |  |  | Others |
| Brenton et al. [14] | 2017 | USA | 36 | 72 |  | 29 |  |  | Breastfeeding |
| Briggs et al. [15] | 2014 | USA | 1023 | 620 |  |  |  |  | Weight |
| Castelo-Branco et al. [16] | 2020 | Sweden | 6602 | 61828 |  |  |  |  | Others |
| Conradi et al. [17] | 2013 | Germany | 245 | 195 | 46.0 (NA) | 181 |  |  | Breastfeeding |
| Dalmay et al. [18] | 1981 | Cuba/Italy/Martinique | 193 | 358 | 40.1 (10.2) | 154 |  | 31.8 (9.6) |  |
| de Jong et al. [19] | 2019 | USA | 151 | 235 | 36.0 (4.5) | 151 (39.1) |  | 39.5 (8.9) | Others |
| De Keyser et al. [20] | 1997 | The Netherlands | 100 | 100 |  |  |  |  | Others |
| Eid et al. [21] | 2022 | Norway | 300 | 77697 |  |  |  |  | Adverse social experiences |
| El-Muzaini et al. [22] | 2020 | Kuwait | 110 | 110 | 34.8 (10.3) | 84 (38.2) |  | 27.3 (9.0) | Tobacco exposure |
| Espinoza-Ramirez et al. [23] | 2014 | Mexico | 83 | 166 |  | 47 |  |  | Previous infections |
| Gardener et al. [24] | 2009 | EUA | 622 | 185103 |  |  |  |  | Tobacco exposure |
| Gatto et al. [25] | 2022 | Iceland | 214 | 27656 | 45.6 (11.1) |  |  |  | Adverse social experiences |
| Goldacre et al. [26] | 2017 | UK | 31 | 164012 |  |  |  |  | Breastfeeding |
| Graves et al. [27] | 2017 | USA | 265 | 412 | 15.7 (NA) | 165 |  | 15.0 (NA) | Breastfeeding |
| Grønning et al. [28] | 1993 | Norway | 136 | 179 |  |  |  |  | Previous infections |
| Gunnarsson et al. [29] | 2015 | UK | 628 | 6187 |  |  |  |  | Weight |
| Gusev et al. [30] | 1996 | Russia | 155 | 112 | 36.3 (9.8) | 96 |  |  | Previous infections |
| Gustavesen et al. [31] | 2014 | Norway | 530 | 918 | 50.5 (12.7) | 391 | 18.2 (11.6) | 32.3 (9.3) | Others |
| Halawani et al. [32] | 2018 | Saudi Arabia | 80 | 160 |  | 52 |  |  | Previous infections |
| Hedstrom et al. [33] | 2015 | Sweden | 1586 | 2800 |  |  |  |  | Weight |
| Hedstrom et al. [34] | 2020 | Sweden | 2522 | 4397 |  |  |  |  | Breastfeeding |
| Hedstrom et al. [35] | 2012 | Sweden | 1571 | 3371 |  |  |  |  | Weight |
| Hedstrom et al. [36] | 2014 | Sweden and USA | 2447 | 2626 |  |  |  |  | Weight |
| Hedstrom et al. [37] | 2015 | Sweden | 6282 | 7924 |  | 4625 |  |  | Epstein-baar virus |
| Hedstrom et al. [38] | 2016 | Sweden | 2455 | 5336 |  |  |  |  | Tobacco exposure |
| Hernàn et al. [39] | 2001 | USA | 292 | 1367 |  |  |  |  | Previous infections |
| Horton et al. [40] | 2022 | USA | 1422 | 1185 |  | 1124 | 17.1 (11.8) |  | Adverse social experiences |
| Hughes et al. [41] | 2020 | Australia | 258 | 508 |  |  |  |  | Epstein-baar virus |
| Jacobs et al.[42] | 2021 | UK | 2250 | 486000 | 55.2 (7.7) | 1635 |  |  | Breastfeeding |
| Krone et al. [43] | 2008 | Germany | 82 | 152 |  |  |  |  | Previous infections |
| Langer-Gould e tal. [44] | 2017 | USA | 519 | 571 |  | 242 |  |  | Breastfeeding |
| Langer-Gould et al. [45] | 2013 | USA | 75 | 913097 |  | 41 |  |  | Weight |
| Leibovitch et al. [46] | 2019 | USA | 32 | 42 | 13.8 (3.9) | 17 |  |  | Epstein-baar virus |
| Magalhaes et al. [47] | 2019 | Canada, Italy and Norway | 2251 | 4028 | 41.9 (10.7) | 1563 | 6.5 (2.8) |  | Epstein-baar virus |
| Majzoobi et al. [48] | 2022 | Iran | 109 | 114 | 10.5 (37.6) | 92 |  |  | Previous infections |
| Mar et al. [49] | 2008 | USA | 312 | 490 | 15.7 (NA) | 277 |  |  | Others |
| McDonald et al. [50] | 2016 | USA | 170 | 331 | 15.2 (3.5) | 107 (62.9) |  |  | Others |
| Mikaeloff et al. [51] | 2009 | France | 137 | 1061 |  |  |  |  | Previous infections |
| Mikaeloff et al. [52] | 2007 | France | 143 | 1122 |  | 93 |  |  | Previous infections |
| Mikaeloff et al. [53] | 2007 | France | 129 | 1038 | 11.5 (3.7) | 83 |  |  | Tobacco exposure |
| Milles et al. [54] | 2021 | France | 60 | 113 |  |  |  |  | Weight |
| Munger et al. [55] | 2013 | USA | 738 | 8401363 |  |  |  |  | Weight |
| Munger et al. [56] | 2011 | USA | 222 | 444 | 23.4 (5.5) |  |  | 28.4 (6.2) | Epstein-baar virus |
| Nasr et al. [57] | 2023 | USA | 490 | 716 |  |  |  |  | Others |
| Nielsen et al. [58] | 2017 | Denmark | 521 | 972 |  | 354 |  |  | Vitamin d |
| Nourbakhsh et al. [59] | 2018 | USA | 356 | 493 | 15.2 (3.2) | 229 | 1.0 (0.9) |  | Epstein-baar virus |
| Oturai et al. [60] | 2021 | Denmark | 919 | 1670 |  |  |  |  | Others |
| Pekmezovic et al. [61] | 2004 | Serbia | 110 | 110 | 34.4 (10.2) | 77 |  |  | Previous infections |
| Pohl et al. [62] | 2006 | Germany | 147 | 147 |  | 98 |  | 12.3 (NA) | Epstein-baar virus |
| Ragnedda et al. [63] | 2015 | Norway | 737 | 1335 |  |  |  |  | Breastfeeding |
| Riise et al. [64] | 2011 | USA | 292 | 68029 |  |  |  |  | Adverse social experiences |
| Sakoda et al. [65] | 2020 | Japan | 103 | 124 | 43.6 (10.9) | 80 | 13.0 (8.6) |  | Breastfeeding |
| Sebastian et al. [66] | 2022 | USA | 534 | 332 |  | 210 (24.2) |  |  | Tobacco exposure |
| Siddiqui et al. [67] | 2021 | Saudi Arabia | 82 | 82 |  | 50 |  |  | Previous infections |
| Smith et al. [68] | 2020 | Sweden | 6109 | 49479 |  |  |  |  | Epstein-baar virus |
| Spitzer et al. [69] | 2013 | Germany | 234 | 885 | 39.7 (7.1) | 171 |  |  | Adverse social experiences |
| Suleiman et al. [70] | 2018 | USA | 326 | 506 | 14.9 (3.2) | 207 |  |  | Tobacco exposure |
| Sullivan et al. [71] | 1984 | USA | 88 | 88 | 42.9 (NA) | 60 |  |  | Others |
| Tarrats et al. [72] | 2002 | Mexico | 94 | 210 | 36.4 (1.2) | 40 |  |  | Others |
| Tremlett et al. [73] | 2016 | Canada | 18 | 17 | 12.5 (4.4) | 10 |  |  | Others |
| Tremlett et al. [74] | 2018 | USA | 151 | 235 |  |  |  |  | Weight |
| Tremlett et al. [75] | 2021 | Canada | 32 | 36 | 16.5 (3.7) | 24 |  | 14.0 (3.9) | Tobacco exposure |
| Ueda et al. [76] | 2014 | Sweden | 459 | 663 |  | 349 |  | 25.1 (4.7) | Breastfeeding |
| Waubant et al. [77] | 2011 | USA | 189 | 66 | 14.9 (3.3) | 124 |  | 12.9 (4.0) | Epstein-baar virus |
| Wesnes et al. [78] | 2015 | Norway and Italy | 1660 | 3050 |  |  |  |  | Weight |
| Wutayd et al. [79] | 2018 | Saudi Arabia | 307 | 307 | 32.9 (8.8) | 230 |  |  | Breastfeeding |
| Xu et al. [80] | 2021 | Sweden | 5867 | 2487113 |  |  |  |  | Epstein-baar virus |
| Xu et al. [81] | 2021 | Sweden | 952 | 743596 |  |  |  |  | Weight |
| Xu et al. [82] | 2021 | Sweden | 4022 | 2418947 |  | 2825 |  |  | Epstein-baar virus |
| Yea et al. [83] | 2013 | Canada | 22 | 77 | 16.1 (1.8) | 16 |  | 13.6 (4.1) | Epstein-baar virus |
| Zaadstra et al. [84] | 2008 | The Netherlands | 2821 | 2550 |  |  |  |  | Epstein-baar virus |
| Zilber et al. [85] | 1996 | Israel | 93 | 94 |  |  |  |  | Vaccines |

**eAppendix 3. Critical appraisal checklist of studies.**

| **Authors** | **Q1** | **Q2** | **Q3** | **Q4** | **Q5** | **Q6** | **Q7** | **Q8** | **Q9** | **Q10** |
| --- | --- | --- | --- | --- | --- | --- | --- | --- | --- | --- |
| Abbasi et al. [1] | NO | NO | YES | NO | YES | YES | YES | YES | UNCLEAR | YES |
| Abdollahpour et al. [2] | YES | YES | YES | YES | YES | YES | YES | YES | UNCLEAR | YES |
| Ahlgren et at. [3] | NO | NO | NO | YES | YES | YES | YES | YES | YES | YES |
| Åkerstedt et al. [4] | UNCLEAR | UNCLEAR | YES | UNCLEAR | YES | NO | NO | YES | UNCLEAR | YES |
| Alkhawajah et al. [5] | YES | YES | YES | YES | YES | YES | YES | YES | YES | YES |
| Aloitabi et al. [6] | YES | YES | NO | YES | YES | YES | YES | YES | NO | YES |
| Ammitzboll et al. [7] | UNCLEAR | UNCLEAR | UNCLEAR | YES | YES | NO | NO | UNCLEAR | UNCLEAR | YES |
| Andersen et al. [8] | NO | NO | NO | YES | YES | YES | YES | YES | YES | YES |
| Bager et al. [9] | UNCLEAR | UNCLEAR | YES | NO | YES | YES | YES | YES | YES | YES |
| Baldin et al. [10] | NO | NO | YES | YES | YES | YES | YES | YES | NO | YES |
| Baldin et al. [11] | UNCLEAR | UNCLEAR | YES | YES | YES | YES | YES | YES | YES | YES |
| Bansil et al. [12] | UNCLEAR | UNCLEAR | UNCLEAR | NO | YES | NO | NO | UNCLEAR | UNCLEAR | YES |
| Bistrom et al. [13] | YES | YES | NO | YES | YES | YES | YES | YES | NO | YES |
| Bourne et al. [14] | NO | NO | YES | YES | YES | YES | YES | YES | NO | YES |
| Brento et al. [15] | YES | YES | UNCLEAR | YES | YES | YES | YES | YES | NO | YES |
| Briggs et al. [16] | NO | NO | YES | YES | YES | YES | YES | YES | YES | YES |
| Castelo-Branco et al. [17] | YES | YES | YES | NO | YES | NO | NO | YES | UNCLEAR | YES |
| Conradi et al. [18] | NO | NO | NO | YES | YES | YES | YES | YES | YES | YES |
| Dalmay et al. [19] | YES | YES | YES | NO | YES | YES | YES | YES | UNCLEAR | YES |
| de Jong et al. [20] | YES | YES | YES | YES | YES | YES | YES | YES | YES | YES |
| De Keyser et al. [21] | UNCLEAR | UNCLEAR | UNCLEAR | NO | YES | NO | NO | YES | UNCLEAR | YES |
| Eid et al. [22] | YES | YES | YES | YES | YES | YES | YES | YES | UNCLEAR | YES |
| El-Muzaini et al. [23] | NO | YES | YES | YES | YES | YES | YES | YES | UNCLEAR | YES |
| Espinoza-Ramirez et al. [24] | YES | YES | YES | YES | YES | YES | YES | YES | YES | YES |
| Gardener et al. [25] | UNCLEAR | UNCLEAR | YES | NO | YES | YES | YES | YES | UNCLEAR | YES |
| Gatto et al. [26] | UNCLEAR | UNCLEAR | YES | YES | YES | YES | YES | NO | YES | YES |
| Goldacre et al. [27] | YES | YES | YES | NO | YES | YES | YES | YES | YES | YES |
| Graves et al. [28] | NO | NO | NO | NO | YES | YES | YES | YES | UNCLEAR | YES |
| Grønning et al. [29] | YES | YES | YES | NO | YES | YES | YES | YES | UNCLEAR | YES |
| Gunnarsson et al. [30] | YES | YES | YES | UNCLEAR | YES | YES | YES | YES | UNCLEAR | YES |
| Gusev et al. [31] | UNCLEAR | UNCLEAR | NO | UNCLEAR | YES | YES | YES | YES | UNCLEAR | YES |
| Gustavesen et al. [32] | NO | NO | NO | NO | YES | YES | YES | YES | NO | YES |
| Halawani et al. [33] | YES | YES | YES | YES | YES | YES | YES | YES | UNCLEAR | YES |
| Hedstrom et al. [34] | YES | YES | YES | NO | YES | YES | YES | YES | YES | YES |
| Hedstrom et al. [35] | YES | YES | YES | YES | YES | YES | YES | YES | YES | YES |
| Hedstrom et al. [36] | YES | YES | YES | NO | YES | YES | YES | YES | NO | YES |
| Hedstrom et al. [37] | YES | YES | YES | YES | YES | YES | YES | YES | UNCLEAR | YES |
| Hedstrom et al. [38] | YES | YES | YES | NO | YES | UNCLEAR | UNCLEAR | YES | UNCLEAR | YES |
| Hedstrom et al. [39] | YES | YES | YES | YES | YES | YES | YES | YES | YES | YES |
| Hernàn et al. [40] | YES | YES | YES | NO | YES | NO | NO | YES | YES | YES |
| Horton et al. [41] | YES | YES | YES | YES | YES | YES | YES | YES | UNCLEAR | YES |
| Hughes et al. [42] | YES | YES | YES | NO | YES | NO | NO | YES | YES | YES |
| Jacobs et al. [43] | YES | YES | YES | NO | YES | YES | YES | YES | NO | YES |
| Krone et al. [44] | YES | YES | YES | YES | YES | NO | NO | YES | UNCLEAR | YES |
| Langer-Gould et al. [45] | YES | YES | YES | YES | YES | YES | YES | YES | UNCLEAR | YES |
| Langer-Gould et al. [46] | NO | NO | NO | YES | YES | YES | YES | YES | UNCLEAR | YES |
| Leibovitch et al. [47] | YES | YES | YES | YES | YES | NO | NO | YES | UNCLEAR | YES |
| Magalhaes et al. [48] | YES | YES | YES | YES | YES | YES | YES | YES | YES | YES |
| Majzoobi et al. [49] | NO | NO | UNCLEAR | YES | YES | NO | NO | NO | UNCLEAR | YES |
| Mar et al. [50] | YES | YES | UNCLEAR | YES | YES | YES | YES | YES | YES | YES |
| McDonald et al. [51] | NO | NO | UNCLEAR | YES | YES | YES | YES | YES | UNCLEAR | YES |
| Mikaeloff et al. [52] | YES | YES | YES | YES | YES | YES | YES | YES | UNCLEAR | YES |
| Mikaeloff et al. [53] | YES | YES | YES | YES | YES | YES | YES | YES | YES | YES |
| Mille et al. [54] | UNCLEAR | UNCLEAR | YES | YES | YES | YES | YES | YES | UNCLEAR | YES |
| Munger et al. [55] | YES | YES | YES | YES | YES | YES | YES | YES | YES | YES |
| Munger et al. [56] | YES | YES | YES | YES | YES | YES | YES | YES | UNCLEAR | YES |
| Nasr et al. [57] | NO | NO | NO | NO | YES | YES | YES | YES | NO | YES |
| Nourbakhsh et al. [58] | NO | NO | NO | YES | YES | YES | YES | YES | UNCLEAR | YES |
| Oturai et al. [59] | YES | YES | YES | YES | YES | YES | YES | YES | YES | YES |
| Pekmezovic et al. [60] | UNCLEAR | UNCLEAR | YES | NO | YES | NO | NO | YES | YES | YES |
| Pohl et al. [61] | UNCLEAR | UNCLEAR | NO | NO | YES | YES | YES | YES | UNCLEAR | YES |
| Ragnedda et al. [62] | YES | YES | YES | NO | YES | YES | YES | YES | UNCLEAR | YES |
| Riise et al. [63] | YES | YES | YES | YES | YES | YES | YES | YES | YES | YES |
| Sakoda et al. [64] | NO | NO | NO | NO | YES | YES | YES | YES | NO | YES |
| Sebastian et al. [65] | YES | YES | UNCLEAR | YES | YES | YES | YES | YES | YES | YES |
| Siddiqui et al. [66] | NO | NO | NO | NO | YES | NO | NO | YES | UNCLEAR | YES |
| Smith et al. [67] | YES | YES | YES | NO | YES | YES | YES | YES | UNCLEAR | YES |
| Spitzer et al. [68] | NO | NO | NO | YES | YES | YES | YES | YES | UNCLEAR | YES |
| Suleiman et al. [69] | YES | YES | NO | NO | YES | YES | YES | YES | UNCLEAR | YES |
| Sullivan et al. [70] | UNCLEAR | UNCLEAR | UNCLEAR | NO | YES | NO | NO | UNCLEAR | UNCLEAR | YES |
| Tarrats et al. [71] | YES | YES | YES | NO | YES | YES | YES | YES | UNCLEAR | YES |
| Tremlett et al. [72] | UNCLEAR | UNCLEAR | NO | NO | YES | YES | YES | YES | UNCLEAR | YES |
| Tremlett et al. [73] | YES | YES | NO | YES | YES | YES | YES | YES | UNCLEAR | YES |
| Tremlett et al. [74] | UNCLEAR | UNCLEAR | YES | UNCLEAR | YES | YES | YES | YES | UNCLEAR | YES |
| Tremlett et al. [75] | NO | NO | YES | UNCLEAR | YES | YES | YES | YES | UNCLEAR | YES |
| Ueda et al. [76] | YES | YES | YES | YES | YES | YES | YES | YES | YES | YES |
| Waubant et al. [77] | YES | YES | NO | YES | YES | YES | YES | YES | UNCLEAR | YES |
| Wesnes et al. [78] | YES | YES | YES | NO | YES | YES | YES | YES | YES | YES |
| Wutayd et al. [79] | YES | YES | YES | NO | YES | YES | YES | YES | NO | YES |
| Xu et al. [80] | NO | NO | YES | YES | YES | YES | YES | YES | UNCLEAR | YES |
| Xu et al. [81] | UNCLEAR | UNCLEAR | YES | YES | YES | YES | YES | YES | UNCLEAR | YES |
| Xu et al. [82] | NO | NO | YES | YES | YES | YES | YES | YES | UNCLEAR | YES |
| Yea et al. [83] | YES | YES | NO | YES | YES | NO | NO | YES | UNCLEAR | YES |
| Zaadstra et al. [84] | YES | YES | UNCLEAR | NO | YES | YES | YES | YES | UNCLEAR | YES |
| Zilber et al. [85] | UNCLEAR | UNCLEAR | YES | YES | YES | NO | NO | UNCLEAR | UNCLEAR | YES |

1. Abbasi M, Nabavi SM, Fereshtehnejad SM, Jou NZ, Ansari I, Shayegannejad V, Mohammadianinejad SE, Farhoudi M, Noorian A, Razazian N, Abedini M, Faraji F. Multiple sclerosis and environmental risk factors: a case-control study in Iran. Neurol Sci. 2017 Nov;38(11):1941-1951. doi: 10.1007/s10072-017-3080-9. Epub 2017 Aug 10. PMID: 28799006.
2. Abdollahpour I, Sormani MP, Nedjat S, Mansournia MA, van der Mei I. The role of nutritional factors during adolescence in multiple sclerosis onset: a population-based incident case-control study. Nutr Neurosci. 2021 Jul;24(7):500-507. doi: 10.1080/1028415X.2019.1647689. Epub 2019 Jul 31. PMID: 31362644.
3. Ahlgren C, Torén K, Odén A, Andersen O. A population-based case-control study on viral infections and vaccinations and subsequent multiple sclerosis risk. Eur J Epidemiol. 2009;24(9):541-52. doi: 10.1007/s10654-009-9367-2. Epub 2009 Jul 26. PMID: 19633994.
4. Åkerstedt T, Olsson T, Alfredsson L, Hedström AK. Insufficient sleep during adolescence and risk of multiple sclerosis: results from a Swedish case-control study. J Neurol Neurosurg Psychiatry. 2023 May;94(5):331-336. doi: 10.1136/jnnp-2022-330123. Epub 2023 Jan 23. PMID: 36690431; PMCID: PMC10176406.
5. Alkhawajah NM, Hussain-Alkhateeb L, Alshamlan YA, Almohaini MO, Aleissa GA, Muayqil TA, Aljarallah S. Shared breastfeeding & other early multiple sclerosis risk factors: A case-control study. Mult Scler Relat Disord. 2021 May;50:102812. doi: 10.1016/j.msard.2021.102812. Epub 2021 Feb 4. PMID: 33581612.
6. Alotaibi S, Kennedy J, Tellier R, Stephens D, Banwell B. Epstein-Barr virus in pediatric multiple sclerosis. JAMA. 2004 Apr 21;291(15):1875-9. doi: 10.1001/jama.291.15.1875. PMID: 15100207.
7. Ammitzboll T, Clausen J. Measles antibody in serum of multiple sclerosis patients, their children, siblings and parents. Acta Neurol Scand. 1972;48(1):47-56. doi: 10.1111/j.1600-0404.1972.tb07526.x. PMID: 5019832.
8. Andersen C, Søndergaard HB, Bang Oturai D, Laursen JH, Gustavsen S, Larsen NK, Magyari M, Just-Østergaard E, Thørner LW, Sellebjerg F, Ullum H, Oturai AB. Alcohol consumption in adolescence is associated with a lower risk of multiple sclerosis in a Danish cohort. Mult Scler. 2019 Oct;25(12):1572-1579. doi: 10.1177/1352458518795418. Epub 2018 Aug 20. PMID: 30124094
9. Bager P, Nielsen NM, Bihrmann K, Frisch M, Hjalgrim H, Wohlfart J, Koch-Henriksen N, Melbye M, Westergaard T. Childhood infections and risk of multiple sclerosis. Brain. 2004 Nov;127(Pt 11):2491-7. doi: 10.1093/brain/awh283. Epub 2004 Sep 15. PMID: 15371288.
10. Baldin E, Daltveit AK, Cortese M, Riise T, Pugliatti M. Exposure to breastfeeding and risk of developing multiple sclerosis. Int J Epidemiol. 2021 May 17;50(2):644-651. doi: 10.1093/ije/dyaa250. PMID: 34000734.
11. Xu Y, Hiyoshi A, Smith KA, Piehl F, Olsson T, Fall K, Montgomery S. Association of Infectious Mononucleosis in Childhood and Adolescence With Risk for a Subsequent Multiple Sclerosis Diagnosis Among Siblings. JAMA Netw Open. 2021 Oct 1;4(10):e2124932. doi: 10.1001/jamanetworkopen.2021.24932. PMID: 34633426; PMCID: PMC8506233.
12. Biström M, Jons D, Engdahl E, Gustafsson R, Huang J, Brenner N, Butt J, Alonso-Magdalena L, Gunnarsson M, Vrethem M, Bender N, Waterboer T, Granåsen G, Olsson T, Kockum I, Andersen O, Fogdell-Hahn A, Sundström P. Epstein-Barr virus infection after adolescence and human herpesvirus 6A as risk factors for multiple sclerosis. Eur J Neurol. 2021 Feb;28(2):579-586. doi: 10.1111/ene.14597. Epub 2020 Nov 22. PMID: 33065762; PMCID: PMC7839468.
13. Bourne T, Waltz M, Casper TC, Kavak K, Aaen G, Belman A, Benson L, Candee M, Chitnis T, Graves J, Greenberg B, Gorman M, Harris Y, Krupp L, Lotze T, Mar S, Ness J, Olsen C, Roalstad S, Rodriguez M, Rose J, Rubin J, Schreiner T, Tillema JM, Kahn I, Waldman A, Barcellos L, Waubant E, Weinstock-Guttman B; US Network of Pediatric MS Centers. Evaluating the association of allergies with multiple sclerosis susceptibility risk and disease activity in a pediatric population. J Neurol Sci. 2017 Apr 15;375:371-375. doi: 10.1016/j.jns.2017.02.041. Epub 2017 Feb 20. PMID: 28320170; PMCID: PMC5606138
14. Brenton JN, Engel CE, Sohn MW, Goldman MD. Breastfeeding During Infancy Is Associated With a Lower Future Risk of Pediatric Multiple Sclerosis. Pediatr Neurol. 2017 Dec;77:67-72. doi: 10.1016/j.pediatrneurol.2017.09.007. Epub 2017 Sep 14. PMID: 29074058.
15. Briggs FB, Acuña BS, Shen L, Bellesis KH, Ramsay PP, Quach H, Bernstein A, Schaefer C, Barcellos LF. Adverse socioeconomic position during the life course is associated with multiple sclerosis. J Epidemiol Community Health. 2014 Jul;68(7):622-9. doi: 10.1136/jech-2013-203184. Epub 2014 Feb 27. PMID: 24577137.
16. Castelo-Branco A, Chiesa F, Conte S, Bengtsson C, Lee S, Minton N, Niemcryk S, Lindholm A, Rosenlund M, Piehl F, Montgomery S. Infections in patients with multiple sclerosis: A national cohort study in Sweden. Mult Scler Relat Disord. 2020 Oct;45:102420. doi: 10.1016/j.msard.2020.102420. Epub 2020 Jul 23. PMID: 32736217.
17. Conradi S, Malzahn U, Paul F, Quill S, Harms L, Then Bergh F, Ditzenbach A, Georgi T, Heuschmann P, Rosche B. Breastfeeding is associated with lower risk for multiple sclerosis. Mult Scler. 2013 Apr;19(5):553-8. doi: 10.1177/1352458512459683. Epub 2012 Sep 4. PMID: 22951352.
18. Dalmay F, Bhalla D, Nicoletti A, Cabrera-Gomez JA, Cabre P, Ruiz F, Druet-Cabanac M, Dumas M, Preux PM. Multiple sclerosis and solar exposure before the age of 15 years: case-control study in Cuba, Martinique and Sicily. Mult Scler. 2010 Aug;16(8):899-908. doi: 10.1177/1352458510366856. Epub 2010 May 12. PMID: 20463038.
19. de Jong HJI, Tremlett H, Zhu F, Ascherio A, Munger KL. Animal exposure over the life-course and risk of multiple sclerosis: A case-control study within two cohorts of US women. Mult Scler Relat Disord. 2019 Jan;27:327-332. doi: 10.1016/j.msard.2018.11.015. Epub 2018 Nov 15. PMID: 30471586.
20. De Keyser J, Zwanikken C. Multiple sclerosis and exposure to house pets during childhood and adolescence: a case-control study. Eur J Neurol. 2021. doi: 10.1111/j.1468-1331.1997.tb00408.x.
21. Eid K, Torkildsen Ø, Aarseth J, Aalstad M, Bhan A, Celius EG, Cortese M, Daltveit AK, Holmøy T, Myhr KM, Riise T, Schüler S, Torkildsen CF, Wergeland S, Gilhus NE, Bjørk MH. Association of adverse childhood experiences with the development of multiple sclerosis. J Neurol Neurosurg Psychiatry. 2022 Jun;93(6):645-650. doi: 10.1136/jnnp-2021-328700. Epub 2022 Apr 4. PMID: 35379699; PMCID: PMC9148981.
22. El-Muzaini H, Akhtar S, Alroughani R. A matched case-control study of risk factors associated with multiple sclerosis in Kuwait. BMC Neurol. 2020 Feb 21;20(1):64. doi: 10.1186/s12883-020-01635-1. Erratum in: BMC Neurol. 2020 Aug 19;20(1):306. doi: 10.1186/s12883-020-01886-y. PMID: 32085743; PMCID: PMC7033919.
23. Espinosa-Ramírez G, Ordoñez G, Flores-Rivera J, Sotelo J. Sunlight exposure and multiple sclerosis in a tropical country. Neurol Res. 2014 Jul;36(7):647-50. doi: 10.1179/1743132813Y.0000000307. Epub 2014 Jan 12. PMID: 24620971.
24. Gardener H, Munger KL, Chitnis T, Michels KB, Spiegelman D, Ascherio A. Prenatal and perinatal factors and risk of multiple sclerosis. Epidemiology. 2009 Jul;20(4):611-8. doi: 10.1097/EDE.0b013e31819ed4b9. PMID: 19333127; PMCID: PMC3132937.
25. Gatto NM, Thordardottir EB, Tomasson G, Rúnarsdóttir H, Song H, Jakobsdóttir J, Aspelund T, Valdimarsdóttir UA, Hauksdóttir A. Association between Adverse Childhood Experiences and Multiple Sclerosis in Icelandic Women-A Population-Based Cohort Study. Brain Sci. 2022 Nov 16;12(11):1559. doi: 10.3390/brainsci12111559. PMID: 36421883; PMCID: PMC9688793.
26. Goldacre A, Pakpoor J, Goldacre M. Maternal and perinatal characteristics of infants who, later in life, developed multiple sclerosis: Record-linkage study. Mult Scler Relat Disord. 2017 Apr;13:98-102. doi: 10.1016/j.msard.2017.02.004. Epub 2017 Feb 4. PMID: 28427711.
27. Graves JS, Chitnis T, Weinstock-Guttman B, Rubin J, Zelikovitch AS, Nourbakhsh B, Simmons T, Waltz M, Casper TC, Waubant E; Network of Pediatric Multiple Sclerosis Centers. Maternal and Perinatal Exposures Are Associated With Risk for Pediatric-Onset Multiple Sclerosis. Pediatrics. 2017 Apr;139(4):e20162838. doi: 10.1542/peds.2016-2838. PMID: 28562303; PMCID: PMC5369674.
28. Grønning M, Riise T, Kvåle G, Albrektsen G, Midgard R, Nyland H. Infections in childhood and adolescence in multiple sclerosis. A case-control study. Neuroepidemiology. 1993;12(2):61-9. doi: 10.1159/000110302. PMID: 8232705.
29. Gunnarsson M, Udumyan R, Bahmanyar S, Nilsagård Y, Montgomery S. Characteristics in childhood and adolescence associated with future multiple sclerosis risk in men: cohort study. Eur J Neurol. 2015 Jul;22(7):1131-7. doi: 10.1111/ene.12718. Epub 2015 Apr 27. PMID: 25919640; PMCID: PMC4975688.
30. Gusev E, Boiko A, Lauer K, Riise T, Deomina T. Environmental risk factors in MS: a case-control study in Moscow. Acta Neurol Scand. 1996 Dec;94(6):386-94. doi: 10.1111/j.1600-0404.1996.tb00050.x. PMID: 9017026.
31. Gustavsen MW, Page CM, Moen SM, Bjølgerud A, Berg-Hansen P, Nygaard GO, Sandvik L, Lie BA, Celius EG, Harbo HF. Environmental exposures and the risk of multiple sclerosis investigated in a Norwegian case-control study. BMC Neurol. 2014 Oct 3;14:196. doi: 10.1186/s12883-014-0196-x. PMID: 25274070; PMCID: PMC4186947.
32. Halawani AT, Zeidan ZA, Kareem AM, Alharthi AA, Almalki HA. Sociodemographic, environmental and lifestyle risk factors for multiple sclerosis development in the Western region of Saudi Arabia. A matched case control study. Saudi Med J. 2018 Aug;39(8):808-814. doi: 10.15537/smj.2018.8.22864. PMID: 30106419; PMCID: PMC6194980.
33. Hedström AK, Olsson T, Alfredsson L. Body mass index during adolescence, rather than childhood, is critical in determining MS risk. Mult Scler. 2016 Jun;22(7):878-83. doi: 10.1177/1352458515603798. Epub 2015 Sep 11. PMID: 26362895.
34. Hedström AK, Adams C, Shao X, Schaefer C, Olsson T, Barcellos LF, Alfredsson L. Breastfeeding is associated with reduced risk of multiple sclerosis in males, predominantly among HLA-DRB1*15:01 carriers. Mult Scler J Exp Transl Clin. 2020 Jun 1;6(2):2055217320928101. doi: 10.1177/2055217320928101. PMID: 32728476; PMCID: PMC7364805.
35. Hedström AK, Olsson T, Alfredsson L. High body mass index before age 20 is associated with increased risk for multiple sclerosis in both men and women. Mult Scler. 2012 Sep;18(9):1334-6. doi: 10.1177/1352458512436596. Epub 2012 Feb 10. PMID: 22328681.
36. Hedström AK, Lima Bomfim I, Hillert J, Olsson T, Alfredsson L. Obesity interacts with infectious mononucleosis in risk of multiple sclerosis. Eur J Neurol. 2015 Mar;22(3):578-e38. doi: 10.1111/ene.12620. Epub 2014 Dec 20. PMID: 25530445; PMCID: PMC4365756.
37. Hedström AK, Lima Bomfim I, Hillert J, Olsson T, Alfredsson L. Obesity interacts with infectious mononucleosis in risk of multiple sclerosis. Eur J Neurol. 2015 Mar;22(3):578-e38. doi: 10.1111/ene.12620. Epub 2014 Dec 20. PMID: 25530445; PMCID: PMC4365756.
38. Hedström AK, Olsson T, Alfredsson L. Smoking is a major preventable risk factor for multiple sclerosis. Mult Scler. 2016 Jul;22(8):1021-6. doi: 10.1177/1352458515609794. Epub 2015 Oct 12. PMID: 26459151.
39. Hernán MA, Zhang SM, Lipworth L, Olek MJ, Ascherio A. Multiple sclerosis and age at infection with common viruses. Epidemiology. 2001 May;12(3):301-6. doi: 10.1097/00001648-200105000-00009. PMID: 11337603.
40. Horton MK, McCurdy S, Shao X, Bellesis K, Chinn T, Schaefer C, Barcellos LF. Case-control study of adverse childhood experiences and multiple sclerosis risk and clinical outcomes. PLoS One. 2022 Jan 13;17(1):e0262093. doi: 10.1371/journal.pone.0262093. PMID: 35025951; PMCID: PMC8757911.
41. Hughes AM, Ponsonby AL, Dear K, Dwyer T, Taylor BV, van der Mei I, Valery PC; Ausimmune Investigator Group; Lucas RM. Childhood infections, vaccinations, and tonsillectomy and risk of first clinical diagnosis of CNS demyelination in the Ausimmune Study. Mult Scler Relat Disord. 2020 Jul;42:102062. doi: 10.1016/j.msard.2020.102062. Epub 2020 Mar 18. PMID: 32305688.
42. Jacobs BM, Noyce AJ, Bestwick J, Belete D, Giovannoni G, Dobson R. Gene-Environment Interactions in Multiple Sclerosis: A UK Biobank Study. Neurol Neuroimmunol Neuroinflamm. 2021 May 28;8(4):e1007. doi: 10.1212/NXI.0000000000001007. PMID: 34049995; PMCID: PMC8192056.
43. Krone B, Pohl D, Rostasy K, Kahler E, Brunner E, Oeffner F, Grange JM, Gärtner J, Hanefeld F. Common infectious agents in multiple sclerosis: a case-control study in children. Mult Scler. 2008 Jan;14(1):136-9. doi: 10.1177/1352458507082069. Epub 2007 Oct 17. PMID: 17942525.
44. Nourbakhsh B, Rutatangwa A, Waltz M, Rensel M, Moodley M, Graves J, Casper TC, Waldman A, Belman A, Greenberg B, Goyal M, Harris Y, Kahn I, Lotze T, Mar S, Schreiner T, Aaen G, Hart J, Ness J, Rubin J, Tillema JM, Krupp L, Gorman M, Benson L, Rodriguez M, Chitnis T, Rose J, Candee M, Weinstock-Guttman B, Shao X, Barcellos L, James J, Waubant E; US Network of Pediatric MS Centers. Heterogeneity in association of remote herpesvirus infections and pediatric MS. Ann Clin Transl Neurol. 2018 Sep 17;5(10):1222-1228. doi: 10.1002/acn3.636. PMID: 30349857; PMCID: PMC6186938.
45. Leibovitch EC, Lin CM, Billioux BJ, Graves J, Waubant E, Jacobson S. Prevalence of salivary human herpesviruses in pediatric multiple sclerosis cases and controls. Mult Scler. 2019 Apr;25(5):644-652. doi: 10.1177/1352458518765654. Epub 2018 Mar 23. PMID: 29569515; PMCID: PMC6119543.
46. Magalhaes S, Pugliatti M, Riise T, Myhr KM, Ciampi A, Bjornevik K, Wolfson C. Shedding light on the link between early life sun exposure and risk of multiple sclerosis: results from the EnvIMS Study. Int J Epidemiol. 2019 Aug 1;48(4):1073-1082. doi: 10.1093/ije/dyy269. PMID: 30561654; PMCID: PMC6693814.
47. Majzoobi MM, Macvandi MR, Basir HG, Sanaei Z, Mazaheri S, Afza M, Arabestani MR. The role of Bordetella pertussis in the development of multiple sclerosis. BMC Neurol. 2022 Mar 1;22(1):70. doi: 10.1186/s12883-022-02606-4. PMID: 35232387; PMCID: PMC8886202.
48. Mar S, Liang S, Waltz M, Casper TC, Goyal M, Greenberg B, Weinstock-Guttman B, Rodriguez M, Aaen G, Belman A, Barcellos LF, Rose J, Gorman M, Benson L, Candee M, Chitnis T, Harris Y, Kahn I, Roalsted S, Hart J, Lotze T, Moodley M, Ness J, Rensel M, Rubin J, Schreiner T, Tillema JM, Waldman A, Krupp L, Graves JS, Waubant E; U.S. Network of Pediatric Multiple Sclerosis Centers. Several household chemical exposures are associated with pediatric-onset multiple sclerosis. Ann Clin Transl Neurol. 2018 Oct 9;5(12):1513-1521. doi: 10.1002/acn3.663. PMID: 30564618; PMCID: PMC6292189.
49. Mar S, Liang S, Waltz M, Casper TC, Goyal M, Greenberg B, Weinstock-Guttman B, Rodriguez M, Aaen G, Belman A, Barcellos LF, Rose J, Gorman M, Benson L, Candee M, Chitnis T, Harris Y, Kahn I, Roalsted S, Hart J, Lotze T, Moodley M, Ness J, Rensel M, Rubin J, Schreiner T, Tillema JM, Waldman A, Krupp L, Graves JS, Waubant E; U.S. Network of Pediatric Multiple Sclerosis Centers. Several household chemical exposures are associated with pediatric-onset multiple sclerosis. Ann Clin Transl Neurol. 2018 Oct 9;5(12):1513-1521. doi: 10.1002/acn3.663. PMID: 30564618; PMCID: PMC6292189.
50. McDonald J, Graves J, Waldman A, Lotze T, Schreiner T, Belman A, Greenberg B, Weinstock-Guttman B, Aaen G, Tillema JM, Hart J, Lulu S, Ness J, Harris Y, Rubin J, Candee M, Krupp LB, Gorman M, Benson L, Rodriguez M, Chitnis T, Mar S, Barcellos LF, Laraia B, Rose J, Roalstad S, Simmons T, Casper TC, Waubant E. A case-control study of dietary salt intake in pediatric-onset multiple sclerosis. Mult Scler Relat Disord. 2016 Mar;6:87-92. doi: 10.1016/j.msard.2016.02.011. Epub 2016 Feb 12. PMID: 27063630; PMCID: PMC4830915.
51. Mikaeloff Y, Caridade G, Suissa S, Tardieu M; KIDSEP Study Group. Clinically observed chickenpox and the risk of childhood-onset multiple sclerosis. Am J Epidemiol. 2009 May 15;169(10):1260-6. doi: 10.1093/aje/kwp039. Epub 2009 Mar 27. PMID: 19329530.
52. Mikaeloff Y, Caridade G, Rossier M, Suissa S, Tardieu M. Hepatitis B vaccination and the risk of childhood-onset multiple sclerosis. Arch Pediatr Adolesc Med. 2007 Dec;161(12):1176-82. doi: 10.1001/archpedi.161.12.1176. PMID: 18056563.
53. Mikaeloff Y, Caridade G, Tardieu M, Suissa S; KIDSEP study group. Parental smoking at home and the risk of childhood-onset multiple sclerosis in children. Brain. 2007 Oct;130(Pt 10):2589-95. doi: 10.1093/brain/awm198. Epub 2007 Sep 7. PMID: 17827175.
54. Milles P, De Filippo G, Maurey H, Tully T, Deiva K; KidBiosep. Obesity in Pediatric-Onset Multiple Sclerosis: A French Cohort Study. Neurol Neuroimmunol Neuroinflamm. 2021 Jul 20;8(5):e1044. doi: 10.1212/NXI.0000000000001044. PMID: 34285094; PMCID: PMC8293287.
55. Munger KL, Bentzen J, Laursen B, Stenager E, Koch-Henriksen N, Sørensen TI, Baker JL. Childhood body mass index and multiple sclerosis risk: a long-term cohort study. Mult Scler. 2013 Sep;19(10):1323-9. doi: 10.1177/1352458513483889. Epub 2013 Apr 2. PMID: 23549432; PMCID: PMC4418015.
56. Munger KL, Levin LI, O'Reilly EJ, Falk KI, Ascherio A. Anti-Epstein-Barr virus antibodies as serological markers of multiple sclerosis: a prospective study among United States military personnel. Mult Scler. 2011 Oct;17(10):1185-93. doi: 10.1177/1352458511408991. Epub 2011 Jun 17. PMID: 21685232; PMCID: PMC3179777.
57. Nasr Z, Schoeps VA, Ziaei A, Virupakshaiah A, Adams C, Casper TC, Waltz M, Rose J, Rodriguez M, Tillema JM, Chitnis T, Graves JS, Benson L, Rensel M, Krupp L, Waldman AT, Weinstock-Guttman B, Lotze T, Greenberg B, Aaen G, Mar S, Schreiner T, Hart J, Simpson-Yap S, Mesaros C, Barcellos LF, Waubant E. Gene-environment interactions increase the risk of paediatric-onset multiple sclerosis associated with household chemical exposures. J Neurol Neurosurg Psychiatry. 2023 Jul;94(7):518-525. doi: 10.1136/jnnp-2022-330713. Epub 2023 Feb 1. PMID: 36725329; PMCID: PMC10272045.
58. Nielsen NM, Munger KL, Koch-Henriksen N, Hougaard DM, Magyari M, Jørgensen KT, Lundqvist M, Simonsen J, Jess T, Cohen A, Stenager E, Ascherio A. Neonatal vitamin D status and risk of multiple sclerosis: A population-based case-control study. Neurology. 2017 Jan 3;88(1):44-51. doi: 10.1212/WNL.0000000000003454. Epub 2016 Nov 30. PMID: 27903815; PMCID: PMC5200855.
59. Nourbakhsh B, Rutatangwa A, Waltz M, Rensel M, Moodley M, Graves J, Casper TC, Waldman A, Belman A, Greenberg B, Goyal M, Harris Y, Kahn I, Lotze T, Mar S, Schreiner T, Aaen G, Hart J, Ness J, Rubin J, Tillema JM, Krupp L, Gorman M, Benson L, Rodriguez M, Chitnis T, Rose J, Candee M, Weinstock-Guttman B, Shao X, Barcellos L, James J, Waubant E; US Network of Pediatric MS Centers. Heterogeneity in association of remote herpesvirus infections and pediatric MS. Ann Clin Transl Neurol. 2018 Sep 17;5(10):1222-1228. doi: 10.1002/acn3.636. PMID: 30349857; PMCID: PMC6186938.
60. Oturai DB, Bach Søndergaard H, Koch-Henriksen N, Andersen C, Laursen JH, Gustavsen S, Kristensen JT, Magyari M, Sørensen PS, Sellebjerg F, Thørner LW, Ullum H, Oturai AB. Exposure to passive smoking during adolescence is associated with an increased risk of developing multiple sclerosis. Mult Scler. 2021 Feb;27(2):188-197. doi: 10.1177/1352458520912500. Epub 2020 Mar 23. PMID: 32202196.
61. Pekmezovic T, Jarebinski M, Drulovic J. Childhood infections as risk factors for multiple sclerosis: Belgrade case-control study. Neuroepidemiology. 2004 Nov-Dec;23(6):285-8. doi: 10.1159/000080094. PMID: 15297795.
62. Pohl D, Krone B, Rostasy K, Kahler E, Brunner E, Lehnert M, Wagner HJ, Gärtner J, Hanefeld F. High seroprevalence of Epstein-Barr virus in children with multiple sclerosis. Neurology. 2006 Dec 12;67(11):2063-5. doi: 10.1212/01.wnl.0000247665.94088.8d. PMID: 17159123.
63. Ragnedda G, Leoni S, Parpinel M, Casetta I, Riise T, Myhr KM, Wolfson C, Pugliatti M. Reduced duration of breastfeeding is associated with a higher risk of multiple sclerosis in both Italian and Norwegian adult males: the EnvIMS study. J Neurol. 2015 May;262(5):1271-7. doi: 10.1007/s00415-015-7704-9. Epub 2015 Mar 21. PMID: 25794863.
64. Riise T, Mohr DC, Munger KL, Rich-Edwards JW, Kawachi I, Ascherio A. Stress and the risk of multiple sclerosis. Neurology. 2011 May 31;76(22):1866-71. doi: 10.1212/WNL.0b013e31821d74c5. PMID: 21624985; PMCID: PMC3115807.
65. Sakoda A, Matsushita T, Nakamura Y, Watanabe M, Shinoda K, Masaki K, Isobe N, Yamasaki R, Kira JI. Environmental risk factors for multiple sclerosis in Japanese people. Mult Scler Relat Disord. 2020 Feb;38:101872. doi: 10.1016/j.msard.2019.101872. Epub 2019 Nov 25. PMID: 31812038.
66. Sebastian P, Cherbuin N, Barcellos LF, Roalstad S, Casper C, Hart J, Aaen GS, Krupp L, Benson L, Gorman M, Candee M, Chitnis T, Goyal M, Greenberg B, Mar S, Rodriguez M, Rubin J, Schreiner T, Waldman A, Weinstock-Guttman B, Graves J, Waubant E, Lucas R; US Network of Pediatric Multiple Sclerosis Centers. Association Between Time Spent Outdoors and Risk of Multiple Sclerosis. Neurology. 2022 Jan 18;98(3):e267-e278. doi: 10.1212/WNL.0000000000013045. Epub 2021 Dec 8. PMID: 34880094; PMCID: PMC8792813.
67. Siddiqui AF, Alsabaani AA, Abouelyazid AY, Wassel YI. Risk factors of multiple sclerosis in Aseer region, Kingdom of Saudi Arabia *A case-control study*. Neurosciences (Riyadh). 2021 Jan;26(1):69-76. doi: 10.17712/nsj.2021.1.20200107. PMID: 33530046; PMCID: PMC8015505.
68. Smith KA, Hiyoshi A, Burkill S, Bahmanyar S, Öckinger J, Alfredsson L, Olsson T, Montgomery S. Hospital diagnosed pneumonia before age 20 years and multiple sclerosis risk. BMJ Neurol Open. 2020 Jun 16;2(1):e000044. doi: 10.1136/bmjno-2020-000044. PMID: 33681783; PMCID: PMC7903180.
69. Spitzer C, Bouchain M, Winkler LY, Wingenfeld K, Gold SM, Grabe HJ, Barnow S, Otte C, Heesen C. Childhood trauma in multiple sclerosis: a case-control study. Psychosom Med. 2012 Apr;74(3):312-8. doi: 10.1097/PSY.0b013e31824c2013. Epub 2012 Mar 9. PMID: 22408134.
70. Suleiman L, Waubant E, Aaen G, Belman A, Benson L, Candee M, Chitnis T, Gorman M, Goyal M, Greenberg B, Harris Y, Hart J, Kahn I, Krupp L, Lotze T, Mar S, Moodley M, Ness J, Nourbakhsh B, Rensel M, Rodriguez M, Rose J, Rubin J, Schreiner T, Tillema JM, Waldman A, Weinstock-Guttman B, Casper TC, Waltz M, Graves JS; Network of Pediatric Multiple Sclerosis Centers.. Early infectious exposures are not associated with increased risk of pediatric-onset multiple sclerosis. Mult Scler Relat Disord. 2018 May;22:103-107. doi: 10.1016/j.msard.2018.03.015. Epub 2018 Mar 26. PMID: 29653437; PMCID: PMC6066281.
71. Sullivan CB, Visscher BR, Detels R. Multiple sclerosis and age at exposure to childhood diseases and animals: cases and their friends. Neurology. 1984 Sep;34(9):1144-8. doi: 10.1212/wnl.34.9.1144. PMID: 6540400.
72. Tarrats R, Ordoñez G, Rios C, Sotelo J. Varicella, ephemeral breastfeeding and eczema as risk factors for multiple sclerosis in Mexicans. Acta Neurol Scand. 2002 Feb;105(2):88-94. doi: 10.1034/j.1600-0404.2002.1o077.x. PMID: 11903117.
73. Tremlett H, Fadrosh DW, Faruqi AA, Zhu F, Hart J, Roalstad S, Graves J, Lynch S, Waubant E; US Network of Pediatric MS Centers. Gut microbiota in early pediatric multiple sclerosis: a case-control study. Eur J Neurol. 2016 Aug;23(8):1308-1321. doi: 10.1111/ene.13026. Epub 2016 May 13. PMID: 27176462; PMCID: PMC4955679.
74. Tremlett H, Zhu F, Ascherio A, Munger KL. Sun exposure over the life course and associations with multiple sclerosis. Neurology. 2018 Apr 3;90(14):e1191-e1199. doi: 10.1212/WNL.0000000000005257. Epub 2018 Mar 7. PMID: 29514944; PMCID: PMC5890609.
75. Tremlett H, Zhu F, Arnold D, Bar-Or A, Bernstein CN, Bonner C, Forbes JD, Graham M, Hart J, Knox NC, Marrie RA, Mirza AI, O'Mahony J, Van Domselaar G, Yeh EA, Zhao Y, Banwell B, Waubant E; US Network of Pediatric MS Centers, the Canadian Pediatric Demyelinating Disease Network. The gut microbiota in pediatric multiple sclerosis and demyelinating syndromes. Ann Clin Transl Neurol. 2021 Dec;8(12):2252-2269. doi: 10.1002/acn3.51476. Epub 2021 Dec 9. PMID: 34889081; PMCID: PMC8670321.
76. Ueda P, Rafatnia F, Bäärnhielm M, Fröbom R, Korzunowicz G, Lönnerbro R, Hedström AK, Eyles D, Olsson T, Alfredsson L. Neonatal vitamin D status and risk of multiple sclerosis. Ann Neurol. 2014 Sep;76(3):338-46. doi: 10.1002/ana.24210. Epub 2014 Jul 10. PMID: 24985080.
77. Waubant E, Mowry EM, Krupp L, Chitnis T, Yeh EA, Kuntz N, Ness J, Chabas D, Strober J, McDonald J, Belman A, Milazzo M, Gorman M, Weinstock-Guttman B, Rodriguez M, Oksenberg JR, James JA; US Pediatric MS Network. Common viruses associated with lower pediatric multiple sclerosis risk. Neurology. 2011 Jun 7;76(23):1989-95. doi: 10.1212/WNL.0b013e31821e552a. PMID: 21646624; PMCID: PMC3109881.
78. Wesnes K, Riise T, Casetta I, Drulovic J, Granieri E, Holmøy T, Kampman MT, Landtblom AM, Lauer K, Lossius A, Magalhaes S, Pekmezovic T, Bjørnevik K, Wolfson C, Pugliatti M, Myhr KM. Body size and the risk of multiple sclerosis in Norway and Italy: the EnvIMS study. Mult Scler. 2015 Apr;21(4):388-95. doi: 10.1177/1352458514546785. Epub 2014 Sep 2. PMID: 25182290.
79. Al Wutayd O, Mohamed AG, Saeedi J, Al Otaibi H, Al Jumah M. Environmental exposures and the risk of multiple sclerosis in Saudi Arabia. BMC Neurol. 2018 Jun 19;18(1):86. doi: 10.1186/s12883-018-1090-8. PMID: 29914402; PMCID: PMC6006694.
80. Xu Y, Hiyoshi A, Smith KA, Piehl F, Olsson T, Fall K, Montgomery S. Association of Infectious Mononucleosis in Childhood and Adolescence With Risk for a Subsequent Multiple Sclerosis Diagnosis Among Siblings. JAMA Netw Open. 2021 Oct 1;4(10):e2124932. doi: 10.1001/jamanetworkopen.2021.24932. PMID: 34633426; PMCID: PMC8506233.
81. Xu Y, Hiyoshi A, Brand JS, Smith KA, Bahmanyar S, Alfredsson L, Olsson T, Montgomery S. Higher body mass index at ages 16 to 20 years is associated with increased risk of a multiple sclerosis diagnosis in subsequent adulthood among men. Mult Scler. 2021 Jan;27(1):147-150. doi: 10.1177/1352458520928061. Epub 2020 Jun 8. PMID: 32507076.
82. Xu Y, Smith KA, Hiyoshi A, Piehl F, Olsson T, Montgomery S. Hospital-diagnosed infections before age 20 and risk of a subsequent multiple sclerosis diagnosis. Brain. 2021 Sep 4;144(8):2390-2400. doi: 10.1093/brain/awab100. PMID: 33693538.
83. Yea C, Tellier R, Chong P, Westmacott G, Marrie RA, Bar-Or A, Banwell B; Canadian Pediatric Demyelinating Disease Network. Epstein-Barr virus in oral shedding of children with multiple sclerosis. Neurology. 2013 Oct 15;81(16):1392-9. doi: 10.1212/WNL.0b013e3182a841e4. Epub 2013 Sep 6. PMID: 24014504; PMCID: PMC3806908
84. Zaadstra BM, Chorus AM, van Buuren S, Kalsbeek H, van Noort JM. Selective association of multiple sclerosis with infectious mononucleosis. Mult Scler. 2008 Apr;14(3):307-13. doi: 10.1177/1352458507084265. Epub 2008 Jan 21. PMID: 18208871.
85. Zilber N, Kahana E. Risk factors for multiple sclerosis: a case-control study in Israel. Acta Neurol Scand. 1996 Dec;94(6):395-403. doi: 10.1111/j.1600-0404.1996.tb00051.x. PMID: 9017027.

**eAppendix 4. PRISMA Checklist.**

| **Section and Topic** | **Item #** | **Checklist item** | **Location where item is reported** |
| --- | --- | --- | --- |
| **TITLE** | | |  |
| Title | 1 | Identify the report as a systematic review. | p. 1 |
| **ABSTRACT** | | |  |
| Abstract | 2 | See the PRISMA 2020 for Abstracts checklist. | pp. 2, 3 |
| **INTRODUCTION** | | |  |
| Rationale | 3 | Describe the rationale for the review in the context of existing knowledge. | pp. 5 - 7 |
| Objectives | 4 | Provide an explicit statement of the objective(s) or question(s) the review addresses. | p. 7 |
| **METHODS** | | |  |
| Eligibility criteria | 5 | Specify the inclusion and exclusion criteria for the review and how studies were grouped for the syntheses. | pp. 8, 9 |
| Information sources | 6 | Specify all databases, registers, websites, organisations, reference lists and other sources searched or consulted to identify studies. Specify the date when each source was last searched or consulted. | p. 8, Figure 1 |
| Search strategy | 7 | Present the full search strategies for all databases, registers and websites, including any filters and limits used. | eAppendix 1 |
| Selection process | 8 | Specify the methods used to decide whether a study met the inclusion criteria of the review, including how many reviewers screened each record and each report retrieved, whether they worked independently, and if applicable, details of automation tools used in the process. | p. 8 |
| Data collection process | 9 | Specify the methods used to collect data from reports, including how many reviewers collected data from each report, whether they worked independently, any processes for obtaining or confirming data from study investigators, and if applicable, details of automation tools used in the process. | p. 9 |
| Data items | 10a | List and define all outcomes for which data were sought. Specify whether all results that were compatible with each outcome domain in each study were sought (e.g. for all measures, time points, analyses), and if not, the methods used to decide which results to collect. | p. 9 |
|  | 10b | List and define all other variables for which data were sought (e.g. participant and intervention characteristics, funding sources). Describe any assumptions made about any missing or unclear information. | p. 9 |
| Study risk of bias assessment | 11 | Specify the methods used to assess risk of bias in the included studies, including details of the tool(s) used, how many reviewers assessed each study and whether they worked independently, and if applicable, details of automation tools used in the process. | p. 10 |
| Effect measures | 12 | Specify for each outcome the effect measure(s) (e.g. risk ratio, mean difference) used in the synthesis or presentation of results. | p. 10 |
| Synthesis methods | 13a | Describe the processes used to decide which studies were eligible for each synthesis (e.g. tabulating the study intervention characteristics and comparing against the planned groups for each synthesis (item #5)). | pp. 9, 10 |
|  | 13b | Describe any methods required to prepare the data for presentation or synthesis, such as handling of missing summary statistics, or data conversions. | pp. 9, 10 |
|  | 13c | Describe any methods used to tabulate or visually display results of individual studies and syntheses. | pp. 9, 10 |
|  | 13d | Describe any methods used to synthesize results and provide a rationale for the choice(s). If meta-analysis was performed, describe the model(s), method(s) to identify the presence and extent of statistical heterogeneity, and software package(s) used. | p. 10 |
|  | 13e | Describe any methods used to explore possible causes of heterogeneity among study results (e.g. subgroup analysis, meta-regression). | p. 10 |
|  | 13f | Describe any sensitivity analyses conducted to assess robustness of the synthesized results. | p. 10 |
| Reporting bias assessment | 14 | Describe any methods used to assess risk of bias due to missing results in a synthesis (arising from reporting biases). | p. 10 |
| Certainty assessment | 15 | Describe any methods used to assess certainty (or confidence) in the body of evidence for an outcome. | pp. 10, 11 |
| **RESULTS** | | |  |
| Study selection | 16a | Describe the results of the search and selection process, from the number of records identified in the search to the number of studies included in the review, ideally using a flow diagram. | p. 11 and Figure 1 |
|  | 16b | Cite studies that might appear to meet the inclusion criteria, but which were excluded, and explain why they were excluded. | p. 11 and Figure 1 |
| Study characteristics | 17 | Cite each included study and present its characteristics. | eAppendix 2 |
| Risk of bias in studies | 18 | Present assessments of risk of bias for each included study. | p. 11 and eAppendix 3 |
| Results of individual studies | 19 | For all outcomes, present, for each study: (a) summary statistics for each group (where appropriate) and (b) an effect estimate and its precision (e.g. confidence/credible interval), ideally using structured tables or plots. | Figures 2 – 5 |
| Results of syntheses | 20a | For each synthesis, briefly summarise the characteristics and risk of bias among contributing studies. | p. 11 |
|  | 20b | Present results of all statistical syntheses conducted. If meta-analysis was done, present for each the summary estimate and its precision (e.g. confidence/credible interval) and measures of statistical heterogeneity. If comparing groups, describe the direction of the effect. | pp. 11 – 13, Figures 2 – 5 |
|  | 20c | Present results of all investigations of possible causes of heterogeneity among study results. | pp. 11 – 13 |
|  | 20d | Present results of all sensitivity analyses conducted to assess the robustness of the synthesized results. | pp. 11 – 13 |
| Reporting biases | 21 | Present assessments of risk of bias due to missing results (arising from reporting biases) for each synthesis assessed. | pp. 11 – 13 |
| Certainty of evidence | 22 | Present assessments of certainty (or confidence) in the body of evidence for each outcome assessed. | pp. 11 – 13, Figures 2 – 5 |
| **DISCUSSION** | | |  |
| Discussion | 23a | Provide a general interpretation of the results in the context of other evidence. | p. 14 |
|  | 23b | Discuss any limitations of the evidence included in the review. | pp. 14 – 17 |
|  | 23c | Discuss any limitations of the review processes used. | pp. 17, 18 |
|  | 23d | Discuss implications of the results for practice, policy, and future research. | pp. 14 – 17 |
| **OTHER INFORMATION** | | |  |
| Registration and protocol | 24a | Provide registration information for the review, including register name and registration number, or state that the review was not registered. | p. 8 |
|  | 24b | Indicate where the review protocol can be accessed, or state that a protocol was not prepared. | p. 8 |
|  | 24c | Describe and explain any amendments to information provided at registration or in the protocol. | NA |
| Support | 25 | Describe sources of financial or non-financial support for the review, and the role of the funders or sponsors in the review. | p. 3 |
| Competing interests | 26 | Declare any competing interests of review authors. | p. 3 |
| Availability of data, code and other materials | 27 | Report which of the following are publicly available and where they can be found: template data collection forms; data extracted from included studies; data used for all analyses; analytic code; any other materials used in the review. | NA |

*From:*  Page MJ, McKenzie JE, Bossuyt PM, Boutron I, Hoffmann TC, Mulrow CD, et al. The PRISMA 2020 statement

**eAppendix 5. Meta-analysis of the association between infections and MS.**

**eAppendix 6. Meta-analysis of the association between immunization and MS.**

**eAppendix 7. Meta-analysis of the association between smoking and MS.**

**eAppendix 8. Meta-analysis of the association between other risk factors and MS.**

**eAppendix 9. Funnel plot and Egger’s test for all analyses.**

1. **EBV**

meta bias, egger random(reml)

Effect-size label: Log odds-ratio

Effect size: _meta_es

Std. err.: _meta_se

Regression-based Egger test for small-study effects

Random-effects model

Method: REML

H0: beta1 = 0; no small-study effects

beta1 = 1.32

SE of beta1 = 1.016

z = 1.30

Prob > |z| = 0.1946

1. **OTHER INFECTIONS**

meta bias, egger random(reml)

Effect-size label: Log odds-ratio

Effect size: _meta_es

Std. err.: _meta_se

Regression-based Egger test for small-study effects

Random-effects model

Method: REML

H0: beta1 = 0; no small-study effects

beta1 = -2.42

SE of beta1 = 0.945

z = -2.56

Prob > |z| = 0.0105

1. **ADVERSE SOCIAL EXPERIENCES**

meta bias, egger random(reml)

Effect-size label: Log odds-ratio

Effect size: _meta_es

Std. err.: _meta_se

Regression-based Egger test for small-study effects

Random-effects model

Method: REML

H0: beta1 = 0; no small-study effects

beta1 = 2.00

SE of beta1 = 2.553

z = 0.78

Prob > |z| = 0.4332

1. **BREASTFEEDING**

meta bias, egger random(reml)

Effect-size label: Log odds-ratio

Effect size: _meta_es

Std. err.: _meta_se

Regression-based Egger test for small-study effects

Random-effects model

Method: REML

H0: beta1 = 0; no small-study effects

beta1 = -1.78

SE of beta1 = 1.433

z = -1.24

Prob > |z| = 0.2138

1. **TOBACCO EXPOSURE**

meta bias, egger random(reml)

Effect-size label: Log odds-ratio

Effect size: _meta_es

Std. err.: _meta_se

Regression-based Egger test for small-study effects

Random-effects model

Method: REML

H0: beta1 = 0; no small-study effects

beta1 = -0.01

SE of beta1 = 1.030

z = -0.01

Prob > |z| = 0.9912

1. **VACCINES**

meta bias, egger random(reml)

Effect-size label: Log odds-ratio

Effect size: _meta_es

Std. err.: _meta_se

Regression-based Egger test for small-study effects

Random-effects model

Method: REML

H0: beta1 = 0; no small-study effects

beta1 = -2.42

SE of beta1 = 0.945

z = -2.56

Prob > |z| = 0.0105

1. **BODY-MASS INDEX**

meta bias, egger random(reml)

Effect-size label: Log odds-ratio

Effect size: _meta_es

Std. err.: _meta_se

Regression-based Egger test for small-study effects

Random-effects model

Method: REML

H0: beta1 = 0; no small-study effects

beta1 = 0.92

SE of beta1 = 0.480

z = 1.92

Prob > |z| = 0.0544

1. **OTHER RISK FACTORS**

meta bias, egger random(reml)

Effect-size label: Log odds-ratio

Effect size: _meta_es

Std. err.: _meta_se

Regression-based Egger test for small-study effects

Random-effects model

Method: REML

H0: beta1 = 0; no small-study effects

beta1 = -0.94

SE of beta1 = 0.904

z = -1.04

Prob > |z| = 0.2993
